# Hepatitis E virus persists in the presence of a type III interferon response

**DOI:** 10.1371/journal.ppat.1006417

**Published:** 2017-05-30

**Authors:** Xin Yin, Xinlei Li, Charuta Ambardekar, Zhimin Hu, Sébastien Lhomme, Zongdi Feng

**Affiliations:** 1 Center for Vaccines and Immunity, The Research Institute at Nationwide Children’s Hospital, Columbus, Ohio, United States of America; 2 Department of Pediatrics, The Ohio State University College of Medicine, Columbus, Ohio, United States of America; University of Southern California, UNITED STATES

## Abstract

The RIG-I-like RNA helicase (RLR)-mediated interferon (IFN) response plays a pivotal role in the hepatic antiviral immunity. The hepatitis A virus (HAV) and the hepatitis C virus (HCV) counter this response by encoding a viral protease that cleaves the mitochondria antiviral signaling protein (MAVS), a common signaling adaptor for RLRs. However, a third hepatotropic RNA virus, the hepatitis E virus (HEV), does not appear to encode a functional protease yet persists in infected cells. We investigated HEV-induced IFN responses in human hepatoma cells and primary human hepatocytes. HEV infection resulted in persistent virus replication despite poor spread. This was companied by a type III IFN response that upregulated multiple IFN-stimulated genes (ISGs), but type I IFNs were barely detected. Blocking type III IFN production or signaling resulted in reduced ISG expression and enhanced HEV replication. Unlike HAV and HCV, HEV did not cleave MAVS; MAVS protein size, mitochondrial localization, and function remained unaltered in HEV-replicating cells. Depletion of MAVS or MDA5, and to a less extent RIG-I, also diminished IFN production and increased HEV replication. Furthermore, persistent activation of the JAK/STAT signaling rendered infected cells refractory to exogenous IFN treatment, and depletion of MAVS or the receptor for type III IFNs restored the IFN responsiveness. Collectively, these results indicate that unlike other hepatotropic RNA viruses, HEV does not target MAVS and its persistence is associated with continuous production of type III IFNs.

## Author summary

HEV infection is a common cause for acute viral hepatitis worldwide. Approximately 20 millions of people are infected annually. In immunocompetent hosts the infection is self-limited and mostly asymptomatic, but the virus frequently persists when immunity is compromised leading to increased risk for cirrhosis. Currently there are no FDA-approved diagnostics or treatments for HEV. Understanding how HEV induces and manipulates host innate immune responses will help elucidate the mechanism(s) of HEV persistence and identify potential targets for therapy. Our results show that unlike other hepatotropic RNA viruses, HEV did not cleave MAVS and stimulated a sustained type III IFN response in persistently infected cells. Furthermore, the JAK/STAT pathway was persistently activated in HEV-replicating cells and responded poorly to exogenously added IFNs. This study uncovers a unique interplay between HEV and the host IFN pathway and provides insight into the mechanism of HEV persistence in patients.

## Introduction

The hepatitis E virus (HEV) causes significant morbidity and mortality worldwide [1, 2]. Although HEV is known for causing acute hepatitis in developing countries, cases of chronic HEV infection have been reported in recent years in industrialized countries in persons with an immune system compromised by treatment with suppressive therapies or HIV co-infection. Patients chronically infected with HEV can rapidly progress to liver fibrosis and cirrhosis if left untreated. The majority of chronic cases are in developed countries and caused by genotype 3 HEV, the most prevalent HEV genotype in those countries. There are no HEV-specific treatments available at present. Ribavirin (RBV) alone or in combination with pegylated-interferon (PegIFN) has been used to treat chronic HEV infection with some success. However, not all patients can be treated with RBV and resistance has been described [3].

Mechanisms for immune control of HEV particularly during chronic infection are poorly understood. The hepatitis C virus (HCV) induces a strong baseline IFN-stimulated gene (ISG) expression that is associated with a persistent infection outcome and poor responsiveness to IFN-based therapy [4, 5]. In contrast, the hepatitis A virus (HAV) does not persist and induces only limited type I IFN responses [6, 7]. Relatively little is known about the IFN response or evasion mechanisms in HEV infection. Elevated ISG expression was detected in patients with chronic HEV infection and HEV-infected mice engrafted with human hepatocytes [8, 9]. In experimentally infected chimpanzees, HEV also induced ISG expression, although the levels were lower than those measured after HCV infection [10]. Interestingly, recent studies have shown that HEV is more resistant to the antiviral effect of IFNs than HCV [11, 12], but the underlying mechanism is not clear.

Despite the differences in early IFN responses and infection outcomes, both HAV and HCV target the mitochondria antiviral signaling protein (MAVS), thereby blocking IFN production in virus-infected cells [13, 14]. A recent study demonstrated that the capacity of HAV to evade MAVS-mediated type I IFN responses defines its host species range [15]. These studies involving HAV and HCV suggest that MAVS inactivation is a requirement for successful infection of the liver by small hepatotropic viruses. Whether HEV also targets MAVS is unknown.

A sole member of the *Hepeviridae* family, HEV has a 7.2 kb single-stranded positive-sense RNA genome encoding three open reading frames (ORF1-3) [16]. ORF1 is a large polyprotein that contains several functional domains essential for virus replication, whereas ORF2 and ORF3 are both translated from a 2.2 subgenomic RNA generated during virus replication and involved in virus assembly and egress, respectively [17, 18]. It has been shown that the putative papain-like protease (PCP) domain and the macro domain of the HEV ORF1 protein block RIG-I and Tank-binding kinase (TBK)-1 ubiquitination, thereby suppressing IFN production [19]. The HEV ORF3 protein, on the other hand, has been reported to enhance IFN production [20]. HEV also induced ISG expression in PLC/PRF/5 human hepatoma cells [21] and in A549 human lung epithelial cells [22]. Relevance of these observations to natural infection is uncertain, however, as most of studies were not conducted in hepatocyte cell lines and/or relied on overexpression of viral proteins.

In this study, we investigated HEV-induced IFN responses in HepG2 human hepatoma cells and primary human hepatocytes. We found that unlike HAV and HCV, HEV does not cleave MAVS, leading to a sustained IFN response in persistently infected cells. Moreover, the JAK/STAT1 pathway was persistently activated and poorly responded to exogenous IFNs, potentially explaining the relative IFN resistance of this virus. These results provide insights into the interactions between HEV and innate signaling during persistence.

## Results

### HEV induces a type III IFN response in human hepatocytes

**Fig 1:**
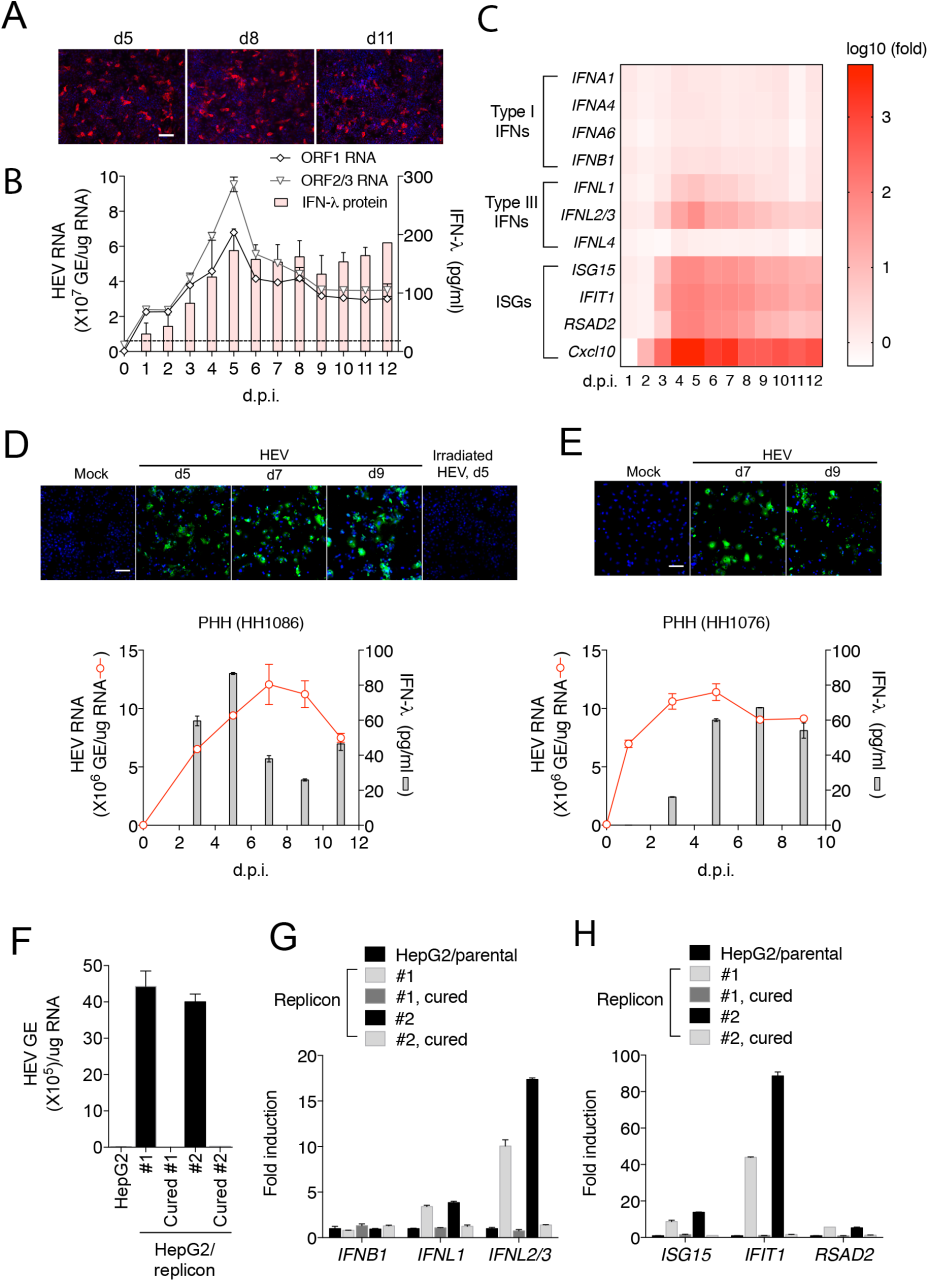
HEV induces a type III-predominant IFN response in human hepatocytes. **(A)** HepG2 cells were inoculated with HEV (1,000 HEV genome equivalents (GE)/cell) and stained with anti-ORF2 antibody at different times after inoculation. Cells were counterstained with DAPI for DNA. Scale bar: 100 μm. **(B)** Kinetics of HEV replication and IFN secretion responses in HepG2 cells. HEV RNA was measured by quantitative RT-PCR using primers targeting either ORF1 (representing the full-length HEV genome) or ORF2/3 (representing the sum of the full-length and the subgenomic HEV RNA). Concentrations of different IFNs (IFN-α, IFN-β, and IFN-λ) in the culture supernatants were measured by specific ELISA. Only IFN-λ protein was detected and, therefore, shown. Dotted line denotes the detection of limit of IFN-λ (15.6 pg/ml). The results show the mean ± SEM of the average of the duplicates in each of 2 independent experiments. (**C**) Kinetics of different IFN and ISG mRNA expression in HEV-infected HepG2 cells. Data are expressed as log_10_ fold change relative to mock-infected cells. **(D-E**) Kinetics of HEV replication and IFN production in HEV-infected primary human hepatocytes (PHHs). PHHs from two different donors (HH1086 and HH1076) were inoculated with HEV (10^4^ GE/cell), and at different days post inoculation stained with chimpanzee immune serum (ch1313) and DAPI (top panels). Cells inoculated with irradiated HEV did not produce positive signal. Scale bar, 100 μm. Intracellular HEV RNA and supernatant IFNs were quantified by qRT-PCR and ELISA, respectively (bottom panels). IFN-α and IFN-β proteins were undetectable. The error bars indicate SEM of results from duplicate wells. **(F)** HEV (ORF1) RNA levels in two independently created clones of HepG2 replicon cells and corresponding replicon-cured cells. The results show the mean ± SEM of 2 independent experiments. **(G-H)** mRNA levels of IFNs and ISGs in HepG2 cells, HepG2 replicon cells and replicon-cured cells. Data are expressed as fold changes relative to the parental cells. The results show the mean ± SEM of 2 independent experiments performed in duplicate each.

To investigate cellular responses to HEV, we used a cell culture-adapted genotype 3 HEV strain (Kernow C1/p6) that replicates efficiently in cell culture [23]. As described elsewhere [23], the particle to FFU ratio of HEV is extremely low (~15,000), and only a small fraction of cells (5–10%) were HEV-positive despite a high dose of inoculum (1x10^3^ HEV genome equivalents per cell). The percentage of HEV-positive cells was stable over 11 days of culture and the majority of HEV foci contained only single infected cells (**Fig 1A**). This suggested establishment of persistent infection, but with poor, if any, virus spread. The percentage of infected cells slightly decreased after 20 days, but the majority remained singly infected (**S1 Fig**). Quantification of HEV ORF1 and ORF2 RNA levels by qRT-PCR showed that HEV replication peaked at around 5 days after infection, then declined to a relatively stable level (**Fig 1B**).

Reduction of HEV RNA replication in HepG2 cells after 5 days may have been caused by activation of the IFN pathways. To test this hypothesis, we measured the concentrations of different types of IFNs in the culture supernatants over the course of HEV infection. Neither IFN-α nor IFN-β proteins were detected. However, IFN-λ protein was readily detected (**Fig 1B**). Persistent HEV replication was observed despite continuous IFN-λ production, even at 30 days after infection (**S1 Fig**). To corroborate this result, we measured the mRNA expression of different types of IFNs (and subtypes for IFN-α) as well as IFN-stimulated genes (ISGs) by RT-qPCR at various times following HEV infection. IFN-λ1 and IFN-λ2/3 were found to be increased at the mRNA levels (**Fig 1C**). IFN-λ4, a recently discovered type III IFN associated with the control of HCV [24], did not increase (**Fig 1C and S2 Fig**). In contrast, little increase in the mRNA levels of IFN-β and multiple IFN-α subtypes was detected, consistent with the absence of IFN-α/β protein production. Increased IFN-λs were associated with increased expression of a number of ISGs (e.g., *ISG15*, *IFIT1*, *RSAD2*, and *CXCL10*). The IFN-λ and ISG response to viral replication peaked at 4–6 days post-inoculation then declined slightly before stabilizing. Importantly, increased IFN-λ, but not IFN-α/β, protein expression was also detected upon HEV infection of primary human hepatocytes (**Fig 1D and 1E**). Similar results were obtained in two independently generated HepG2 cell clones harboring an HEV subgenomic replicon RNA (**Fig 1F–1H**). Replication of HEV RNA was required for the induction of IFNs and ISGs since their expression was reduced to a basal level when the replicon was eliminated by treatment with IFN-α and ribavirin. Collectively, these results demonstrate that HEV persisted in the cells despite the continuous production of IFN-λs.

### Type III IFNs exert an antiviral effect against HEV

**Fig 2:**
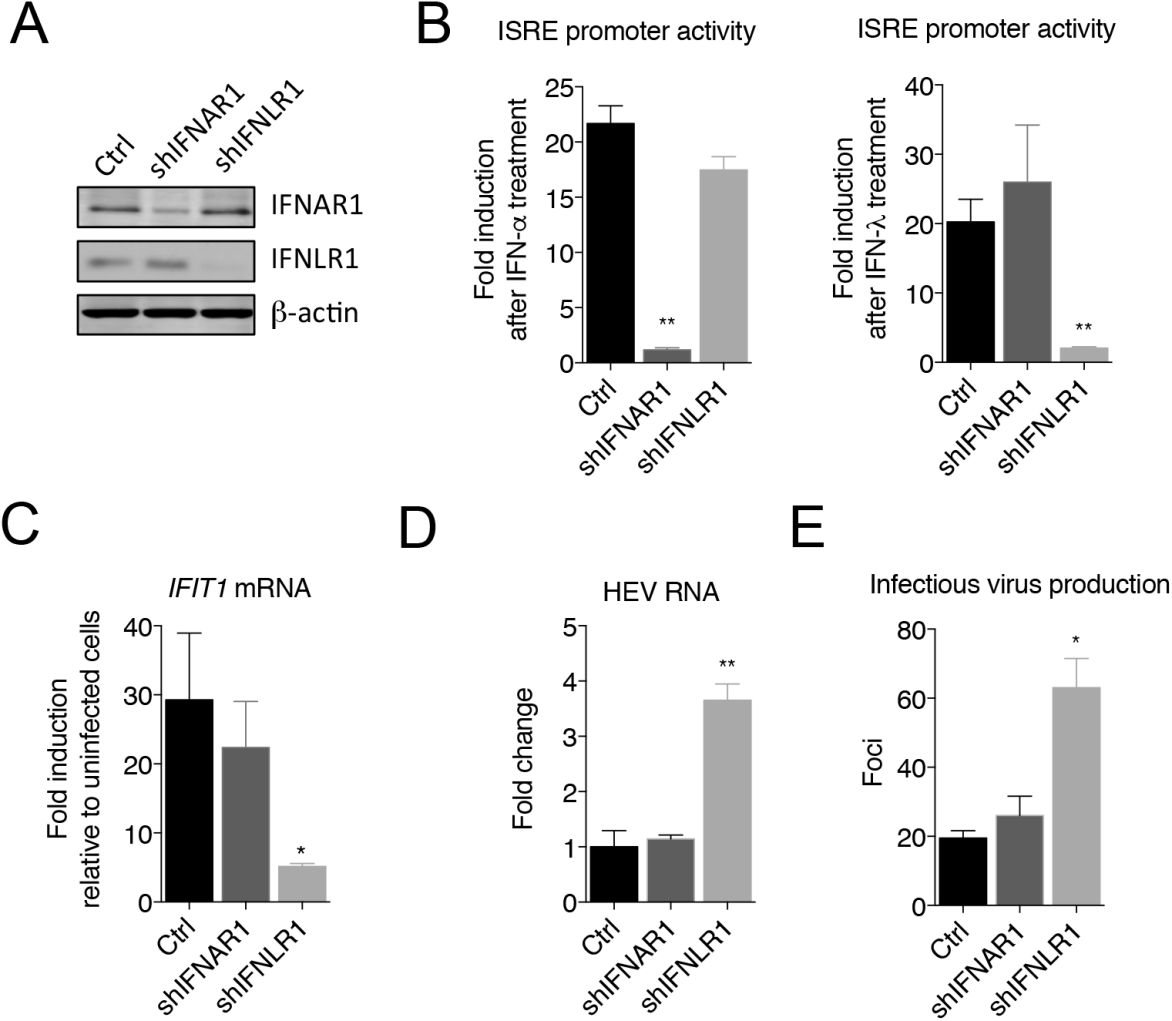
Type III IFN response regulates HEV replication. **(A)** Immunoblots of IFNAR1, IFNLR1 and β-actin in HepG2 cells transduced with lentiviruses expressing gene-specific shRNA or GFP (Ctrl). **(B)** ISRE promoter activity in different cells after IFN-α or IFN-λ treatment. HepG2 cells were transfected with an interferon-sensitive response element (ISRE) promoter-driven firefly luciferase reporter plasmid ISRE-Luc and a herpes simplex virus thymidine kinase promoter-driven Renilla luciferase (TK-RLuc) plasmid for 24 h, then treated with IFN-α (100ng/ml) or IFN-λ1 (220ng/ml) for 24 h. Data are expressed as fold changes relative to non-treated cells based on the relative luciferase activities (firefly luciferase vs. Renilla luciferase). Shown are representative results (mean ± SEM) from two independent experiments each performed in triplicate. **(C-E)** Effects of IFN receptor knockdown on ISG expression and HEV replication. HepG2 cells transduced with lentiviruses expressing gene-specific shRNA or GFP (Ctrl) were infected with HEV for 5 days. (**C**) Intracellular IFIT1 mRNA expression determined by qRT-PCR. Results are represented as fold changes relative to HEV-infected control cells. (**D**) HEV RNA abundance determined by qRT-PCR (fold changes relative to HEV-infected control cells). (**E**) Cells expressing different shRNA or GFP (Ctrl) were infected with HEV for 5 days, harvested and subjected to three rounds of freeze-thaws prior to inoculation to naïve HepG2 cells. Infected cells were detected by IFA and HEV foci were counted after 5 days. Each data point represents the mean ± SEM of at least 2 independent experiments in duplicate each. * P<0.05; ** P<0.01.

To assess the role of released IFN-λs in regulating HEV replication, we depleted IFNLR1, a component of the type III IFN receptor, by transducing HepG2 cells with a lentivirus expressing an *IFNLR1-*specific short hairpin RNA (shRNA). For comparison, we also depleted IFNAR1, receptor for type I IFNs. The knockdown efficiency was examined by western blotting (**Fig 2A**). Depletion of these receptors greatly reduced the cellular responsiveness to IFN-α and IFN-λ, respectively (**Fig 2B**). Notably, the magnitude of HEV-induced ISG expression was significantly reduced in cells depleted of IFNLR1 when compared to the parental cells or cells depleted of IFNAR1 (**Fig 2C**). Moreover, depletion of IFNLR1, but not IFNAR1, resulted in a 3-fold increase in the HEV RNA abundance and infectious virus production **(Fig 2D and 2E**), suggesting that HEV replication is restricted by the type III IFNs induced by infection.

### HEV-induced type III IFN production is dependent on both RIG-I and MDA5

**Fig 3:**
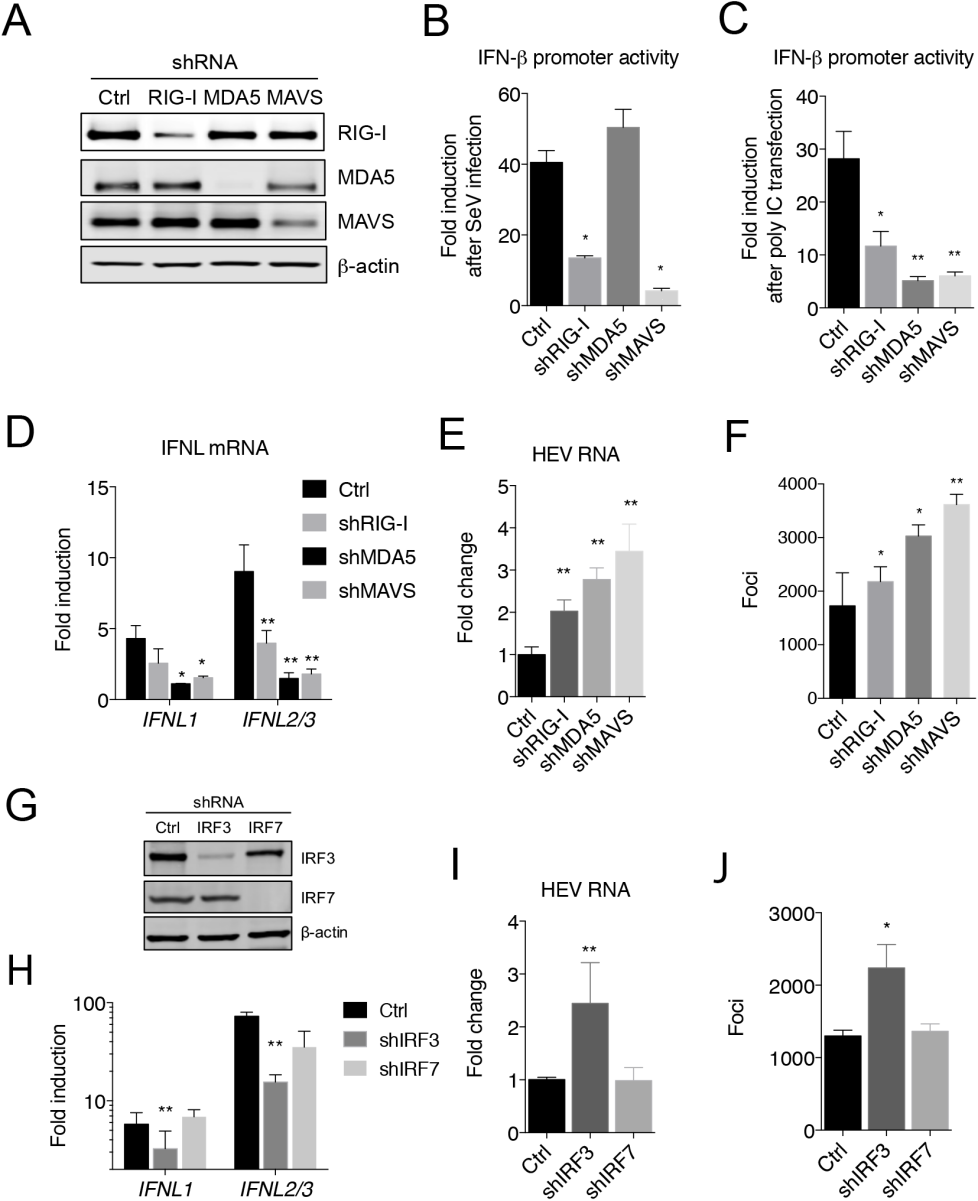
Signaling pathways involved in HEV-induced IFN response. Immunoblots of RIG-I, MDA5, MAVS and β-actin in HepG2 cells expressing gene-specific shRNA, or GFP (Ctrl). **(B-C)** IFN-β promoter activity in HepG2 cells with different gene knockdown following Sendai virus (SeV) infection (B) or poly IC transfection (C). Cells were transfected with IFN-β-Luc and TK-RLuc (for normalization of transfection efficiency) 20 h prior to SeV infection or poly IC transfection. Cells were lysed and luciferase activity was determined 20 h after SeV infection or 12 h after poly IC transfection. Data are presented as fold changes relative to non-treated cells. Shown are representative results from two independent experiments each performed in triplicate. **(D-F)** Effect of RIG-I, MDA5 or MAVS knockdown on HEV replication and host IFN responses. Control and shRNA-expressing HepG2 cells were inoculated with HEV (1000 GE/cell). IFN-λ mRNA expression (D), HEV RNA abundance (E), and HEV-positive foci (F) were determined at 5 days after infection. The results show the mean ± SEM of 2 independent experiments performed in duplicate each. * P<0.05; ** P<0.01. **(G)** Immunoblots of IRF-3, IRF-7 and β-actin in HepG2 cells transduced with lentiviruses expressing GFP (Ctrl) or gene-specific shRNA. (**H-J**) Effect of IRF-3 or IRF-7 knockdown on HEV replication and IFN responses. Control and shRNA-expressing cells were inoculated with HEV (1,000 GE/cell). IFN-λ mRNA expression (H), HEV RNA abundance (I), and HEV-positive foci (J) in different cells were determined after 5 days. The results show the mean ± SEM of 2 independent experiments each performed in duplicate wells. * P<0.05; ** P<0.01. Scale bar (upper panel in F), 100 μm.

Both genomic and subgenomic HEV RNAs are capped and polyadenylated [25], raising the question of how HEV is detected by the cells. To determine the signaling pathway(s) involved in type III IFN production by HEV, we depleted cytoplasmic RNA sensors, namely RIG-I and MDA5, as well as their downstream adaptor protein MAVS by transducing HepG2 cells with lentiviruses expressing gene-specific shRNA. Western blots showed that the respective protein levels in the transduced cells were substantially reduced when compared to control cells transduced with a lentivirus expressing GFP (**Fig 3A**). Cells were then challenged with Sendai virus (SeV), a potent agonist for RIG-I, or poly IC, which primarily stimulates MDA5 when delivered intracellularly by transfection. Depletion of RIG-I led to a substantial reduction in the IFN response to SeV, and to a lesser degree poly IC, whereas depletion of MDA5 reduced the cellular response to poly IC, but not to SeV, confirming the functional knockdown of respective innate sensing pathways (**Fig 3B and 3C**). As expected, depletion of MAVS also reduced the IFN response. Upon HEV infection, cells depleted of MDA5, MAVS, and to a lesser degree RIG-I, displayed reduced type III IFN production (**Fig 3D**), suggesting that both RIG-I and MDA5 are involved in HEV-induced IFN response. Notably, the level of intracellular HEV RNA and the number of infected cells were both increased in cells depleted of MDA5, MAVS, and to a less degree RIG-I (**Fig 3E and 3F)**. Depletion of RIG-I, MDA5, or MAVS in the replicon cells also resulted in increased HEV RNA levels (**S3 Fig**).

Transcription factors interferon regulatory factor (IRF)-3 and IRF-7 have been implicated for the induction of IFN-λ [26]. Depletion of IRF-3, but not IRF-7, resulted in a similar reduction in IFN-λ mRNA expression in HEV-infected cells (**Fig 3G and 3H**). Accordingly, HEV replication was increased in IRF-3-depleted cells, but not in IRF-7 depleted cells (**Fig 3I and 3J**). IRF-3 was found in the nucleus of ~5% of HEV-infected cells, but in 0.1% of uninfected cells (**S4 Fig**), indicating that IFN-λ was produced primarily, if not exclusively, from HEV-infected cells.

Taken together, these data demonstrated that both RIG-I and MDA5 participated in sensing HEV genomes, resulting in a MAVS-dependent type III IFN response.

### HEV does not target MAVS

**Fig 4:**
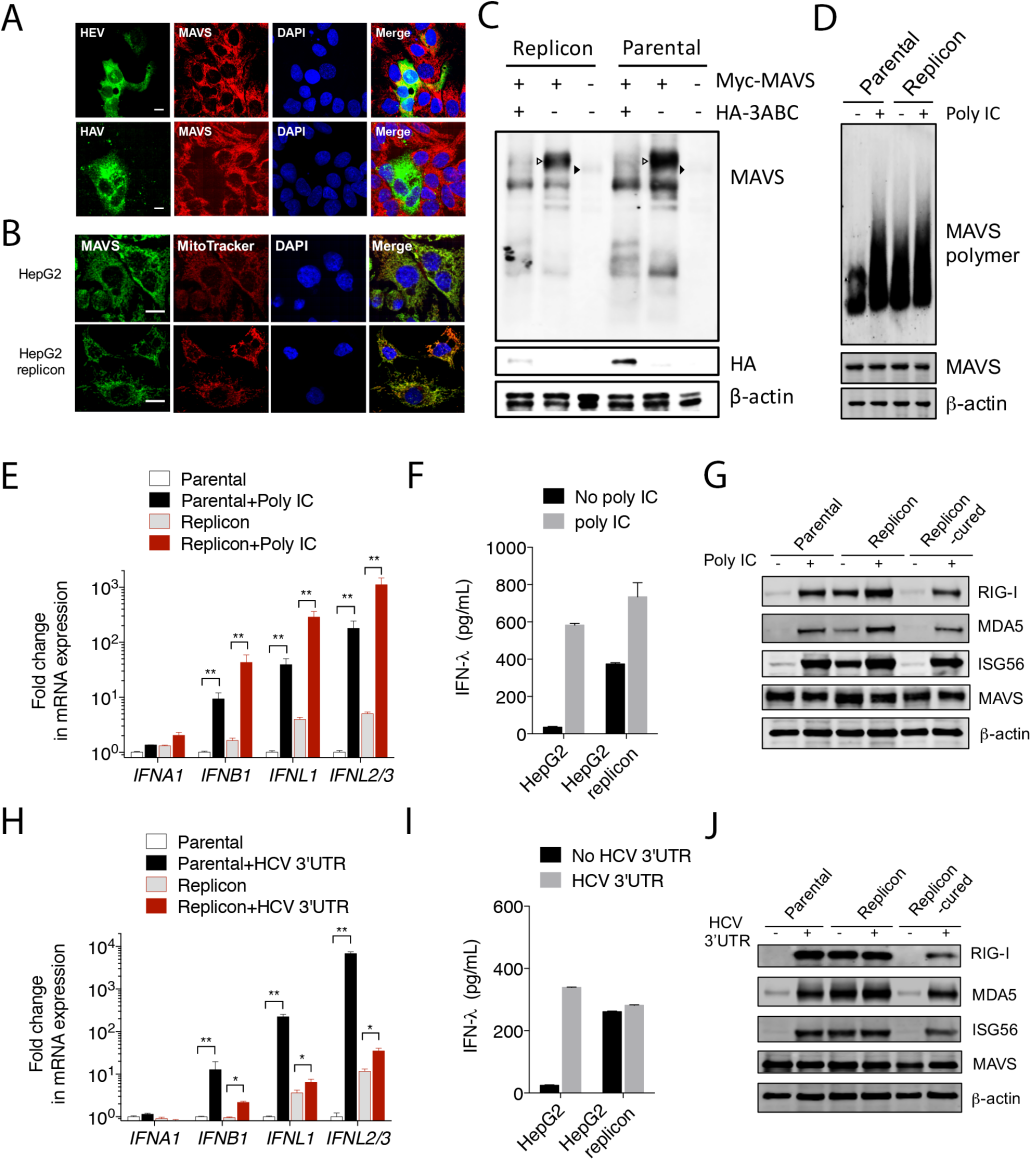
HEV does not target MAVS. **(A)** Confocal images showing MAVS and viral antigens in HepG2 cells infected with either HEV (top) or HAV (bottom). Cells were stained 5 days after infection with a rabbit anti-MAVS, chimpanzee 1313 serum (HEV), or a murine monoclonal antibody K24F2 (HAV). DAPI was used to stain the nuclei. Scale bar: 10 μm. **(B)** Confocal images showing the mitochondrial localization of MAVS in HepG2 cells with or without HEV replicon. MAVS was stained with a rabbit antibody against MAVS (green). Mitochondria was visualized with MitoTracker (red). Nuclei were stained with DAPI. Scale bar: 10 μm. **(C)** HepG2 cells with or without the HEV replicon were transfected with a MAVS-expressing plasmid along with a HAV 3ABC-expressing plasmid or an empty vector. The endogenous (closed arrowheads) and overexpressed MAVS (open arrowheads) were detected with a rabbit anti-MAVS antibody. Note that co-expression of HAV 3ABC led to the degradation of MAVS. (**D**) HepG2 cells with or without the HEV replicon were transfected with poly IC (6 μg/ml). After 6 h, cells were lysed and subjected to Western blot analysis using antibodies against MAVS and β-actin. Crude mitochondria were isolated and subjected to SDD-AGE for detection of MAVS polymer. **(E-F)** HepG2 cells with or without the HEV replicon were transfected with poly IC (6 μg/ml). After 6 h, intracellular IFN mRNA levels were measured by qRT-PCR (E) and supernatant IFN-λ concentration was measured by ELISA (F). In panel E, data are expressed as fold changes relative to mock transfected cells containing no replicon, and the results show the mean ± SEM of 2 independent experiments. **(G)** Immunoblots of endogenous RIG-I, MDA5, ISG56, MAVS, and β-actin in HepG2 cells, replicon cells or replicon-cured cells following poly IC transfection (1.5 μg/ml, 12 h). **(H-I)** HepG2 cells with or without the HEV replicon were transfected with the hepatitis C virus (HCV) 3’UTR RNA. After 6 h, intracellular IFN mRNA levels were measured by qRT-PCR (H), and concentrations of supernatant IFN-λ were measured by ELISA (I). The results show the mean ± SEM of 2 independent experiments. **(J)** Immunoblots of endogenous RIG-I, MDA5, ISG56, MAVS, and β-actin in HepG2 cells with or without the replicon before or after HCV 3’UTR RNA transfection (3.6 μg/ml, 14 h).

The critical dependence of HEV-induced IFN response on MAVS indicated that the function of MAVS remained intact in HEV-infected cells. To test this, we determined the MAVS protein abundance in cells infected with either HEV or HAV. MAVS was largely absent in HAV-infected cells, as expected. However, its expression was not reduced in cells infected with HEV (**Fig 4A**), and its mitochondrial localization was not altered in cells harboring the HEV replicon (**Fig 4B**). A small fraction of MAVS colocalized with peroxisomes. However, no gross difference in this rare colocalization was found between the parental and the replicon cells (**S5 Fig**). In addition, the MAVS protein size was identical between cells with or without HEV replicon (**Fig 4C**). These data indicate that MAVS is not degraded by HEV.

Upon activation, MAVS forms “prion-like” polymers [27]. In HepG2 cells, MAVS polymers only became detectable following poly IC transfection. However, MAVS polymers were present in the replicon cells even without poly IC transfection, consistent with elevated ISG expression in these cells. Transfection with poly IC in the replicon cells further increased the amount of MAVS polymers (**Fig 4D**), indicating that a portion of MAVS were not polymerized and capable of responding to stimulation.

### HEV inhibits RIG-I-, but not MDA5-mediated signaling

The above analyses suggested that MAVS is activated to mediate IFN-λ production in HEV-infected cells. To further investigate the impact of HEV on MAVS-mediated signaling, we compared cells with or without the HEV replicon for their responsiveness to poly IC (an MDA5 agonist), or HCV 3’ untranslated region (UTR) RNA, an agonist of RIG-I [28]. Although unstimulated replicon cells had a higher baseline IFN response than the parental cells, poly IC transfection stimulated IFN expression to a similar extent in both (**Fig 4E**). Likewise, more IFN-λ proteins were released from the replicon cells during the 6 h period of poly IC treatment (**Fig 4F**). Moreover, Western blots revealed that the protein levels of several ISGs including RIG-I, MDA5 and ISG56 were higher in the replicon cells than in the parental cells or in the replicon cured cells, and their expression was further increased following poly IC stimulation (**Fig 4G**). However, the HCV 3’UTR RNA failed to induce more IFNs in the replicon cells (**Fig 4H**) and a similar amount of IFN-λ protein was released from HepG2 cells and HepG2/replicon cells during the 6 h period of treatment (**Fig 4I**). Moreover, the protein levels of ISGs (RIG-I, MDA5, and ISG56), which were expressed at higher levels in unstimulated replicon cells, remained unchanged following HCV 3’UTR RNA stimulation (**Fig 4J**). The poor response to HCV 3’UTR RNA in the replicon cells was not due to impaired transfection since the same method was used for poly IC transfection. Similar results were obtained when cells were infected with SeV, which also stimulates the RIG-I pathway (**S6 Fig**). These results demonstrated that MAVS remained functionally intact in the HEV-replicating cells, and that HEV specifically blocks RIG-I signaling, likely at a step(s) upstream of MAVS.

### HEV-induced STAT1 phosphorylation renders infected cells refractory to exogenous IFNs

**Fig 5:**
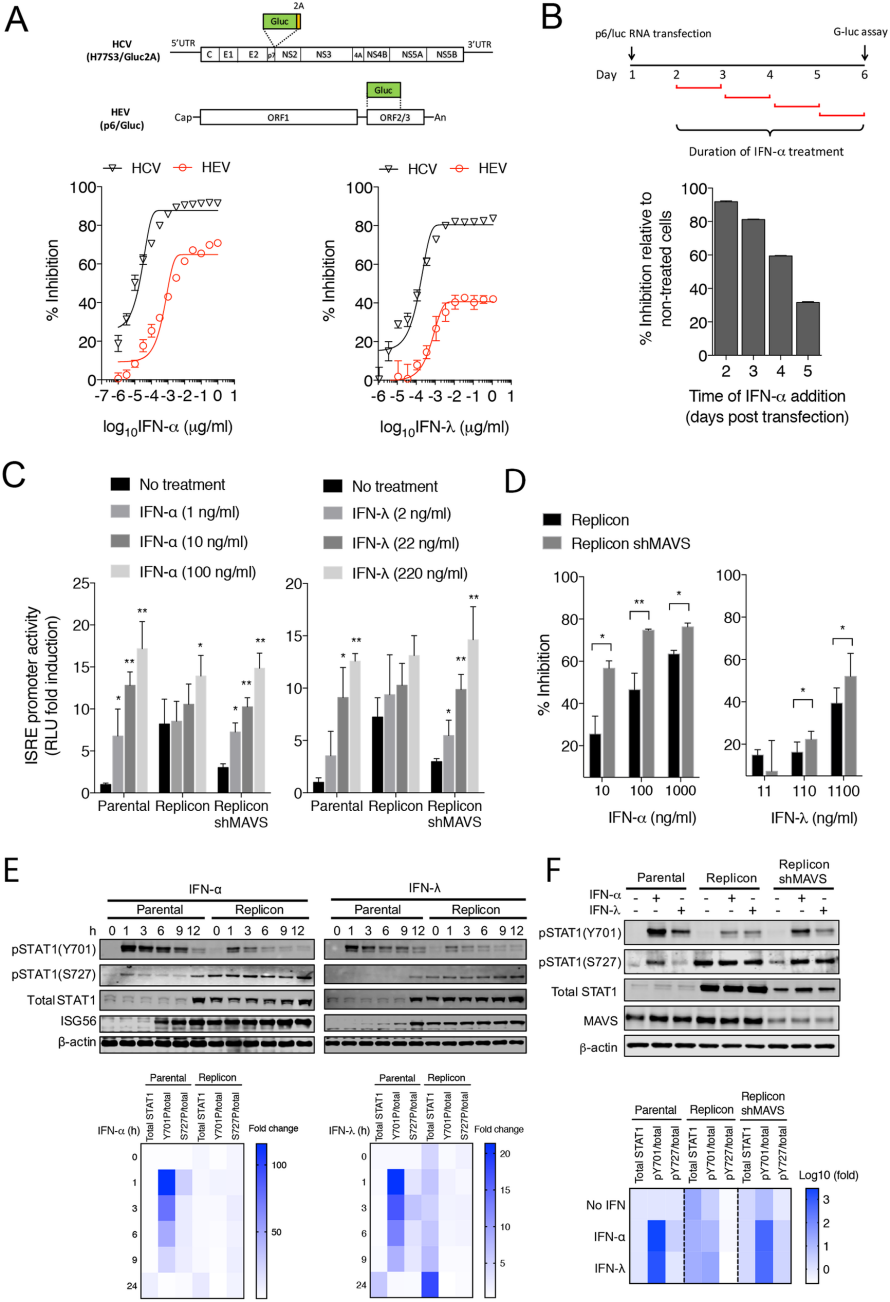
HEV-infected cells are refractory to exogenous IFNs due to basal activation of the JAK/STAT signaling. **(A)** Comparison of antiviral effect of IFN-α and IFN-λ on HCV and HEV replication. Top, a schematic diagram of HCV and HEV constructs used in the experiment. Bottom, Huh-7 cells were transfected with HCV (H77S3/Gluc2A) or HEV (Kernow-C1 p6/luc) RNA. After 24 h, cells were treated with IFN-α or IFN-λ at indicated concentrations. Supernatant Gluc activity was measured 4 days after transfection. Data were presented as percent inhibition relative to the non-treated controls. Shown are the representative results from 2 independent experiments each performed in triplicates. Error bars denote S.E.M. **(B)** Huh-7 cells were transfected with HEV (p6/luc) RNA and treated with IFN-α (100 ng/ml) at indicated times. Data were presented as percent inhibition relative to the non-treated controls. **(C)** Different HepG2 cell lines were transfected with ISRE-luc and TK-RLuc (an internal control for transfection efficiency). 24 h post transfection, cells were treated with IFN-α (100 ng/ml) or IFN-λ1 (220 ng/ml). Cells were harvested 24 h later for luciferase assays. Data are expressed as fold changes relative to non-treated cells based on the relative luciferase activities (firefly luciferase vs. renilla luciferase). The results show the mean ± SEM of 2 independent experiments. (**D**) Antiviral effects of IFN-α and IFN-λ in the HepG2 replicon cells with or without MAVS. Cells were treated with indicated doses of recombinant IFN-α or IFN-λ proteins. After 3 days, intracellular RNA levels were measured by qRT-PCR using primers targeting HEV ORF1. The results (mean ± SEM of 2 independent experiments) show the percentage of inhibition in HEV replication relative to non-treated cells. * P<0.05; ** P<0.01. (**E**) Immunoblots of total and phosphorylated STAT1, ISG56, and β-actin protein levels in different HepG2 cells treated with recombinant IFN-α (100 ng/ml) or IFN-λ (220 ng/ml) for various times. Lower panels show fold changes in total STAT1 proteins and pSTAT1/total STAT1 ratios relative to untreated parental cells. (**F**) Immunoblots of total and phosphorylated STAT1, MAVS, and β-actin protein levels in different HepG2 cells treated with IFN-α (100 ng/ml) or IFN-λ (220 ng/ml) for 1 h. Lower panel shows fold changes in total STAT1 protein levels and pSTAT1/total STAT1 ratios relative to untreated parental cells.

HEV is considered relatively resistant to exogenous IFN treatment when compared to HCV [11, 12]. The mechanism for the IFN resistance is currently not understood. We obtained similar results when recombinant IFNs were added one day after transfection of viral RNA (**Fig 5A**). The antiviral effect of IFNs waned when added at later times (**Fig 5B**), suggesting that HEV replication became relatively resistant to IFNs once virus replication has been established.

Although type I and type III IFNs utilize different receptors, both signal through the JAK/STAT pathway [29]. To address the question of whether constant activation of the JAK/STAT signaling renders the infected cells more resistant to exogenous IFNs, a luciferase reporter driven by an IFN-stimulated response element (ISRE) promoter was used. Activity of the ISRE was measured in cells with or without an HEV replicon after treatment with recombinant IFN-α or IFN-λ. The basal level of luciferase expression was higher (~6-fold) in the replicon cells, consistent with an elevated IFN response in these cells. Both IFN-α and IFN-λ dose-dependently induced luciferase expression in cells without replicon, ranging from 4–16 fold (**Fig 5C**). By comparison, the ISRE reporter gene response increased less than 2-fold in replicon cells treated with the highest dose of IFNs, indicating an establishment of IFN resistance in these cells. Induction of several ISGs (e.g., *ISG15* and *IFIT1*) by IFN-α or IFN-λ was also impaired in the replicon cells (**S7 Fig**). Importantly, the responsiveness to IFNs was largely restored after MAVS or IFNLR1 depletion (**Fig 5C and S8 Fig**), suggesting that HEV-induced activation of the endogenous IFN pathways plays a critical role in developing resistance to further IFN treatment. Consistent with this, MAVS depletion also enhanced the antiviral effects of IFN-α or IFN-λ in the replicon cells (**Fig 5D**).

Both tyrosine phosphorylation and serine phosphorylation of STAT1 is required for its maximal activity [30]. Whereas phosphorylation at the tyrosine 701 (pY701) is essential for efficient DNA binding, phosphorylation at the serine 727 (pS727) is thought to augment ISG expression in a gene-specific manner [30, 31]. To further investigate the mechanism underlying HEV-induced IFN refractoriness, we compared the levels of the total and phosphorylated STAT1 proteins in cells with or without HEV replicon either before or after IFN treatment. The basal level of the total STAT1 protein was much higher in the replicon cells than in the parental cells (**Fig 5E and 5F**), consistent with STAT1 itself being an ISG. In addition, the basal level of pS727, and to a lesser extent pY701, were also higher in the replicon cells. Treatment with IFNs led to a significant increase in the levels of both pY701 and pS727 in the parental cells. However, the increase was much less in the replicon cells, despite higher levels of the total STAT1 proteins (**Fig 5F**). A close examination of STAT1 nuclear/cytoplasmic distribution revealed that although high levels of pS727 were present in the replicon cells, the majority of it retained in the cytoplasm and failed to translocate into the nucleus even after high dose IFN treatment (**S9 Fig**). IFN-induced nuclear translocation of pY701 appeared to be normal in the replicon cells, but the extent of phosphorylation was much less than in the parental cells since the level of the total STAT1 proteins was much higher in the replicon cells. Notably, MAVS depletion led to a reduced basal level of total and phosphorylated STAT1, and IFN-induced STAT1 phosphorylation was largely restored (**Fig 5G**). Since MAVS depletion led to a 3-fold increase in the HEV RNA abundance (**S3 Fig**), this result indicated that HEV does not interfere with the JAK/STAT1 signaling directly.

## Discussion

The purpose of this study was to compare host IFN responses against HEV with those elicited by other hepatitis viruses that persist (HCV) or not (HAV). Both HAV and HCV target MAVS for proteolysis, suggesting that this mechanism for inactivating the IFN response is generally important for infection of the liver by hepatotropic viruses. Our studies with HEV indicate this is not the case. Several lines of evidence indicate that HEV does not target MAVS. First, MAVS abundance, protein size as well as its mitochondria localization were not altered in HEV-infected cells or in cells harboring an HEV replicon. Second, HEV-infected cells and replicon cells produced a sustained IFN response in a MAVS-dependent manner. Third, MAVS-mediated MDA5 signaling remained intact in the replicon cells. Lastly, MAVS was required for elevated STAT1 phosphorylation in the replicon cells. These results provide strong evidence that in contrast to HAV and HCV, HEV neither cleaves MAVS nor interferes with its function.

The lack of MAVS cleavage in infected cells is likely due to an absence of HEV protease activity directed against this signaling protein. Although the HEV ORF1 protein contains a putative papain-like cysteine protease (PCP) domain, convincing evidence for protease activity is lacking. Proteases encoded by positive-stranded RNA viruses are generally involved in the processing of viral polyproteins. However, while apparently truncation of HEV ORF1 proteins was observed in some early studies [32–34], only full-length ORF1 proteins have been detected using robust mammalian overexpression systems and HEV replicon cells [35, 36]. ORF1 may be present at low abundance in infected cells and development of high quality antibodies against ORF1 will likely be needed to firmly address if the PCP domain possesses protease activity towards the HEV polyprotein and cellular substrates.

Consistent with the preservation of MAVS function, HepG2 cells containing the HEV replicon produced a sustained type III IFN response, as evidenced by the elevated expression of IFN-λs and multiple ISGs. Why only type III, but not type I IFNs were produced is not clear, but similar results were reported in studies with hepatitis B virus (HBV) and HCV [37–42]. Interestingly, polymorphisms in type III IFNs have been linked to HCV clearance [43], suggesting that type III IFNs play a critical role in regulating antiviral responses in liver. Since the receptors for type I IFNs are broadly expressed while the receptors for type III IFNs are restricted to epithelial cells such as hepatocytes, one hypothesis is that hepatocytes mainly produce type III IFNs in response to infections to limit host antiviral responses to only locally infected cells.

Another finding from this study is that the activation of the IFN pathway rendered HEV-infected cells more resistant to further stimulation with exogenous IFNs. Our results suggest that this is likely due to persistent activation of the JAK/STAT1 signaling, rather than virus-mediated inhibition. Both the total and phosphorylated STAT1 proteins were elevated in cells harboring the HEV replicon, but STAT1 phosphorylation was minimally increased after treatment with exogenous IFN-α or IFN-λ. Furthermore, although present at a high level in the replicon cells, serine phosphorylated STAT1 (pS727) primarily located in the cytoplasm and did not enter the nucleus even after treatment with high doses of IFN-α or IFN-λ. Thus, these cells became highly resistant to both type I and type III IFNs (likely type II IFN as well) provided exogenously. Such a strategy likely favors the virus *in vivo*, where infected cells are exposed to IFNs produced from different cell types (e.g. plasmacytoid dendritic cells, natural killer cells, and T cells) [44, 45]. Importantly, MAVS depletion effectively reversed the IFN responsiveness of these cells and enhanced the antiviral effects of IFNs, indicating a host feedback mechanism rather than a direct viral antagonism is responsible. Several ISGs, such as USP18 and members of the suppressor of cell signaling (SOCS), have been shown to negatively regulate JAK/STAT activity [46, 47]. USP18 was significantly increased in HEV-infected cells. However, SOCSs were not (**S10 Fig**). While additional work is needed to elucidate the mechanism for HEV-induced IFN resistance, this data may provide an explanation for the relative resistance of HEV to exogenous IFN treatment observed in this and two other recent studies [11, 12].

Despite the elevated expression of IFN-λs and ISGs, the IFN level was insufficient to eliminate HEV and the virus persisted in culture. Elimination of HEV was possible, but only when cells were treated with high doses of IFNs for an extended period (**S11 Fig**). How HEV is able to replicate in the presence of multiple ISGs remains a question for future studies. It is worth noting that certain ISGs (e.g., ISG15, PKR, and ADAR) facilitate virus replication by countering the antiviral actions of IFNs [48, 49]. For example, ISG15 is a well-known factor that is associated with IFN resistance in hepatitis C patients [50], and was highly induced in HEV-infected cells as well as in HEV-infected patients and chimpanzees [9, 10]. More work is needed to define the roles of different ISGs in the life cycle of HEV.

This study sheds new light on HEV persistence. HEV infection is typically self-limited, but it frequently establishes persistence when the host immune system is compromised. Elevated ISG expression has been detected in both acute and chronic HEV infections [9, 10], as well as in HEV infected humanized chimeric mice where no human immune cells are involved [8]. Thus, the type III IFNs produced by HEV infected cells could be an important source of IFNs that drive ISG expression *in vivo*, although involvement of other cell types cannot be ruled out. In the case of HAV and HCV, pDCs are recruited to infected liver and produce copious IFNs after contacting infected cells [51–53]. pDCs may be similarly activated in HEV infection and contribute to the overall IFN response. In this regard, creation of an IFN refractory state in infected cells would favor persistent HEV replication. Thus, the production of type III IFNs by HEV infected cells may be essential for HEV persistence in immunosuppressed patients where adaptive immunity is compromised and the virus is not cleared from persistently infected cells.

In summary, we have shown that HEV induced a sustained type III IFN response in infected cells. This is in sharp contrast to HAV and HCV, both of which cleave MAVS and ablate IFN production in cells they infect. We show that although HEV induced-type III IFNs restricted HEV replication, the IFN level was insufficient to eliminate the virus. Instead, it rendered infected cells refractory to high doses of exogenous IFNs. Our data provide insight into the mechanisms of HEV persistence and the relative IFN resistance of this virus.

## Materials and methods

### Cells and viruses

Huh-7 cells (a gift from Stanley Lemon at the University of North Carolina) and HepG2 cells (CRL-10741, ATCC) were maintained at 37°C in 5% CO_2_ in Dulbecco Modified Eagle medium (DMEM) containing 10% fetal bovine serum (FBS), 100 U/ml penicillin and 100 μg/ml streptomycin. Cryopreserved primary human hepatocytes were purchased from the In Vitro ADMET Laboratories (Columbia, MD) and maintained according to provider’s instructions. HepG2 cells harboring HEV replicon p6/neo, HepG2/p6neo, were generated by transfecting HepG2 cells with in vitro transcribed p6/neo RNA, and selected with 700 μg/ml G418 sulfate (Invivogen) starting 3 days post-transfection. To eliminate the HEV replicon, HepG2/p6neo cells were treatment with IFN-α (100 ng/ml, Sigma) and ribavirin (10 μM, Sigma) for 5 weeks. These resulting cells (replicon-cured) did not contain detectable HEV RNA. Stable short hairpin RNA (shRNA) knockdown cells were generated by transducing cells with shRNA-expressing lentiviral particles (MISSION shRNA lentiviral system, Sigma), and selected with 2 ug/ml puromycin (Invivogen). Pooled cells were used in all experiments. The target sequences are the following: RIG-I: CCAGAATTATCCCAACCGATA; MDA5: CCAACAAAGAAGCAGTGTATA; MAVS: GCATCTCTTCAATACCCTTCA; IRF-3: GCCAACCTGGAAGAGGAATTT; IRF-7: CCCGAGCTGCACGTTCCTATA; IFNAR1: GCTCTCCCGTTTGTCATTTAT; IFNLR1: CCCTAGTTAGGCCCAGATAAA.

HEV stock was generated by transfecting Huh-7 cells with in vitro transcribed HEV Kernow C1/p6 RNA, as previously described [54]. Hepatitis A virus stock (HM175/18f) and hepatitis C virus infectious clone (H77S3/Gluc) were kindly provided by Stanley Lemon (University of North Carolina at Chapel Hill). Sendai virus (Cantell strain) was purchased from Charles River Laboratories.

### Plasmids, reagents and antibodies

The infectious cDNA clone of the HEV genotype 3 Kernow-C1 p6 strain, was kindly provided by Suzanne Emerson (National Institutes of Health, Bethesda, MD) [23, 55]. The HEV Kernow-C1 p6 replicon construct harboring a neomycin resistant gene (*neo*) was constructed by overlapping polymerase chain reaction (PCR). The fragments from nt 4767 to 5359 of the HEV p6 genome and the full-length *neo* gene were amplified from p6 construct and pcDNA3.1 (Invitrogen) by using the following primer pairs: AflII-p6-F (5'-CACCCTTAAGGGTTTCTGGAAGAAGCATTCTG-3') and M-p6/neo-R (5'-CACCCTTAAGGGTTTCTGGAAGAAGCATTCTG-3'), as well as M-p6/neo-F (5´-TGTTTGTTGCATCGCCCATTGGATCACCATGATTGAACAAGATGGATTGCA-3´) and HpaI-p6/neo-R (5´-CACCGTTAAC TCAGAAGAACTCGTCAAGAAGGCGAT-3´). The resulting PCR fragments were joined and cloned into the intermediate plasmid pMD18T-p6/3´ which was generated by cloning the fragment of HEV kernow-C1/p6 from nt 4767 to the 3′ end into pMD18-T Simple vector (Clontech), yielding pMD18T-p6/3´-neo. The fragment digested from this construct with AflII and HindIII was subsequently cloned into the parental p6 backbone, yielding plasmid p6/neo. Reporter plasmids IFN-β-luc (firefly luciferase under the human IFN-β promoter), TK-RLuc (Renilla luciferase under the human thymidine kinase promoter), ISRE-luc (firefly luciferase under the human IFN-stimulated responsive element promoter) were kindly provided by Stanley Lemon (University of North Carolina, Chapel Hill).

High molecular weight (HMW) poly IC was obtained from InvivoGen and reconstituted in PBS at 5 mg/ml. HepG2 cells were transfected with poly IC using DMRIE-C or Lipofectamine 3000 reagent (Invitrogen) for 6 h unless otherwise indicated. HCV 3'-UTR RNA was kindly provided by Takeshi Saito (University of Southern California). HepG2 cells were transfected with 3.6 μg/ml of HCV 3'-UTR RNA using the Lipofectamine 3000 reagent. IFNL4-Halo and Halo-control plasmids as well as recombinant human IFN-λ4 were kindly provided by Ludmila Prokunina-Olsson (National Institutes of Health, Bethesda, MD). Recombinant human IFN-α2a (Sigma, H6041) was used to treat cells for 24 h at 100 ng/ml unless otherwise indicated. Human IL-29/IFN Lambda 1 (11725–1) was purchased from PBL and was used to treat cells for 24 h at 200 ng/ml unless otherwise indicated.

Chimpanzee anti-HEV convalescent-phase serum (ch1313) was kindly provided by Suzanne Emerson (National Institutes of Health, Bethesda, MD). Rabbit anti-pORF2 antibody was a gift from XJ Meng (Virginia Tech). Mouse monoclonal antibody K24F2 to HAV was a gift from Stanley Lemon (University of North Carolina, Chapel Hill). Other antibodies were obtained from: RIG-I (Enzo, ALX-210-932-C100), MDA5 (Enzo, ALX-210-935-C100), MAVS (Enzo, ALX-210-929-C100), HA (Sigma, H9658), PMP70 (Sigma, SAB4200181), IFN-λ4 (Millipore, MABF227), STAT1 (Cell Signaling, 14994S), pSTAT1(Ser727) (Cell Signaling, 8826 and 9177S), pSTAT1(Tyr701) (Cell Signaling, 9167S), IRF3 (Cell Signaling, 11904S), IRF-7 (Santa Cruz, sc-74472), Sendai virus (MBL, PD029), ISG56 (Thermo, PA3-848), IFNLR1(R&D, AF5260-SP), IFNAR1 (Santa Cruz, SC9391), SOCS1 (Cell Signaling, 3950T), SOCS2 (Cell Signaling, 2779T), SOCS3 (Cell Signaling, 2932T), USP18 (Cell Signaling, 4813), Lamin A/C (Cell Signaling, 2032S), GAPDH (Millipore, MAB374), and β-actin (Sigma, A2228).

### Real-time qRT-PCR

Total RNA was extracted from HepG2 cells with the RNeasy Kit (Qiagen) in accordance with the manufacturer’s instructions. Real-time qRT-PCR was performed to quantify the HEV RNA with the iTaq Universal Probes One-Step kit (Bio-Rad) using the primer pair that specifically target ORF2 gene: forward primer: HEV-F (5´-GGTGGTTTCTGGGGTGAC-3´), reverse primer: HEV-R (5’- AGGGGTTGGTTGGATGAA-3´), and probe: HEV-P (5´-FAM-TGATTCTCAGCCCTTCGC–TAMRA-3´) or the primer pair that specifically target ORF1 gene: forward primer: p6/ORF1-F (5´- AAGACCTTCTGCGCTTTGTT-3´), reverse primer: p6/ORF1-R (5’- TGACTCCTCATAAGCATCGC-3´), and probe: p6/ORF1-P (5´-FAM- CCGTGGTTCCGTGCCATTGA–TAMRA-3´). A synthetic full-length HEV Kernow C1/p6 RNA was used as standards.

The endogenous IFN and ISG expression levels were measured by real-time RT-PCR using an iTaq Universal SYBR Green One-Step Kit (Bio-Rad) with specific primers: IFN-α, [56] [42], IFN-λ1, IFN-λ2/3 [42, 57], IFN-λ4 [58], CXCL10, GAPDH [42], IFN-β [42] [59], ISG56 [59], ISG15, RSAD2 [39], USP18 [60], SOCS1, SOCS2 and SOCS-3 [60]. The mRNA levels of glyceraldehyde-3-phosphate dehydrogenase (GAPDH) were determined in the same samples for normalization. ΔΔCT was used to calculate the fold changes relative to the controls [61]. The detailed primer sequences are provided in the supplementary S1 Table.

### Semidenaturing detergent agarose gel electrophoresis (SDD-AGE)

SDD-AGE was performed as described previously [27]. Briefly, crude mitochondria isolated from cells transfected with or without poly IC were resuspended in 1x sample buffer (0.5× TBE, 10% glycerol, 2% SDS, and 0.0025% bromophenol blue), loaded onto a 1.5% agarose gel (Bio-Rad) in the running buffer (1x TBE and 0.1% SDS), and subjected to electrophoresis for 2 h with a constant voltage of 100 V at 4°C. The proteins were transferred to a polyvinylidene difluoride (PVDF) membrane followed by immunoblotting with a rabbit antibody to MAVS.

### Immunoblotting

Cellular lysates were collected on ice in the lysis buffer (100 mM Tris-HCl (pH 7.5), 50 mM NaCl, 5 mM EDTA, and 1% Triton X-100) in the presence of protease inhibitor cocktail (Roche). Samples were separated by SDS-PAGE, and transferred to a PVDF membrane (Bio-rad). Membranes were incubated overnight at 4°C with primary antibody diluted in the Odyssey® Blocking Buffer (LI-COR Biosciences). After washing with PBS-T for three times, the membranes were incubated for 1 h with the appropriate secondary antibodies. Protein bands were detected with an Odyssey Infrared Imaging System (LI-COR Biosciences). For the detection of IFNLR1, for which appropriated secondary antibodies were not available at LI-COR, membranes were wetted with SuperSignal West Pico Chemiluminescent Substrate (Thermo) and exposed to X-ray film (RPI).

### Indirect immunofluorescence assay (IFA) and confocal microscopy

HepG2 cells (4×10^4^) were seeded onto eight-well Lab-Tek II CC^2^ slides (Nunc) one day before infection. IFA for detection of HEV-infected cells was performed as described [62]. To examine the subcellular localization of MAVS and IRF3, cells were fixed with 4% paraformaldehyde for 20 minutes and permeabilized with 0.2% Triton X-100 for 15 minutes. Cells were stained with pre-absorbed ch1313 serum and a rabbit anti-MAVS, mouse anti-PMP70, or rabbit anti-IRF3 for 1 h, and subsequently incubated with Alexa Fluor 488/594-conjugated goat-anti-rabbit IgG, Alexa Fluor 488/594-conjugated goat-anti-mouse IgG, or Alexa Fluor 488-conjugated goat-anti-human IgG (Invitrogen) for 1 h. After adding antifade-4 6-diamidino-2-phenylindole (DAPI) mounting solution (Sigma), slides were viewed with a Zeiss LSM 510 confocal microscope with a 63x (NA1.2) apochromatic water objective. Images were acquired using the ZEN 2009 software.

### Promoter reporter assay

Cells in 96-well plates were transfected with IFN-β-Luc (100 ng/well) or ISRE-Luc (100 ng/well), together with TK-RLuc (10 ng/well) by using the TransIT-X2 Dynamic Delivery System (Mirus Bio). Transfected cells were then infected with 100 hemagglutinin units/ml of Sendai virus for 20 h or treated with recombinant IFN-α or IFN λ1 for 24 h at the indicated concentrations. Luminescence assays were performed in opaque 96-well plates with a Dual-Luciferase Reporter Assay System (Promega) according to the manufacturer’s instructions. Luminescence was measured using a FLUOStar Optima (BMG Labtech) plate reader. Each experiment was performed in triplicate wells.

### Antiviral activity of IFNs

The full-length HCV construct containing a Gaussia luciferease (Gluc) gene inserted between core and p7 (H77S3/Gluc2A) was linearized with XbaI and subjected to in vitro transcription using the T7 In Vitro Transcription kit (Ambion). The subgenomic HEV construct containing a Gluc gene, kindly provided by Suzanne Emerson (NIH, Bethesda), was linearized with MluI and subjected to in vitro transcription using the mMachine mMessenger Transcription kit (Ambion). Huh-7 cells were transfected with in vitro transcribed viral RNA using the TransIT mRNA transfection reagent (Mirus Bio). One day after transfection, cells were split into 96-well plates and treated with IFN-α or IFN-λ at indicated concentrations. In the time-of-addition experiment, IFN-α was added at different days after transfection and replaced with fresh medium on the following days. Luciferase activity in the culture supernatant was measured on day 5 after transfection by a *Gaussia* luciferase kit (Promega).

### ELISA

Supernatant IFN-α, IFN-β or IFN-λ concentrations were measured by the human IFN-α (41100), human IFN-β (41410) or IFN-λ (61840) ELISA kits (PBL Interferon Resources, Piscasaway, NJ) following manufacturer’s instructions.

### Statistical analysis

Values are shown as mean ± SD. Statistical significance between groups was determined with unpaired student’s t-test using GraphPad Prism 6.0 (GraphPad, San Diego, CA).

## Supporting information

S1 Table List of primers used in quantitative RT-PCR. (DOCX)

S1 Fig Long-term HEV replication and IFN-λ production in HepG2 Cells. (DOCX)

S2 Fig Lack of IFN-λ4 protein production in HepG2 cells and HepG2/replicon cells. (DOCX)

S3 Fig Effects of RIG-I, MDA5, or MAVS knockdown on HEV replication in HepG2/replicon cells. (DOCX)

S4 Fig IRF-3 nuclear translocation in HEV-infected HepG2 cells. (DOCX)

S5 Fig Colocalization of MAVS and PMP70 in HepG2 cells and HepG2/replicon cells. (DOCX)

S6 Fig Impaired RIG-I signaling in the HepG2/replicon cells. (DOCX)

S7 Fig Effect of HEV on IFN-induced ISG expression. (DOCX)

S8 Fig IFNLR1 depletion restores IFN-α-induced STAT1 phosphorylation in the HepG2 replicon cells. (DOCX)

S9 Fig Distribution of STAT1 and pSTAT1 between cytosol and nucleus in different HepG2 cells. (DOCX)

S10 Fig SOCS1-3 and USP18 expression in HEV infected cells and in the replicon cells. (DOCX)

S11 Fig Elimination of HEV replicon RNA following prolonged treatment with high doses of IFNs. (DOCX)
