# Supplementary material for: Hepatitis E virus persists in the presence of a type III interferon response
Source: PLoS Pathog. 2017 May 30;13(5):e1006417. doi: 10.1371/journal.ppat.1006417 (PMC5466342; doi:10.1371/journal.ppat.1006417)
Supplement: S1 Table — (DOCX) [file ppat.1006417.s001.docx]

S1 Table. Primers used for qRT-PCR

| Gene | Forward (5’→3’) | Reverse (5’→3’) |
| --- | --- | --- |
| *IFNA1* | GCCTCGCCCTTTGCTTTACT | CTGTGGGTCTCAGGGAGATCA |
| *IFNA4* | ACCTGGTTCAACATGGAAATG | ACCAAGCTTCTTCACACTGCT |
| *IFNA6* | TCCATGAGGTGATTCAGCAGAC | GCTGCTGGTAAAGTTCAGTATAGAGTTT |
| *IFNB1* | GTGCCTGGACCATAGTCAGAGTGG | TGTCCAGTCCCAGAGGCACAGG |
| *IFNL1* | CGCCTTGGAAGAGTCACTCA | GAAGCCTCAGGTCCCAATTC |
| *IFNL2 and IFNL3* | AGTTCCGGGCCTGTATCCAG | GAGCCGGTACAGCCAATGGT |
| *IFNL4* | CGATCCTGGAGCTGCTG | TTTGTGACGCCTCTTCTGG |
| *CXCL10* | GTGGCATTCAAGGAGTACCTC | GCCTTCGATTCTGGATTCAGACA |
| *ISG15* | GGCTGGGAGCTGACGGTGAAG | GCTCCGCCCGCCAGGCTCTGT |
| *RSAD2* | TTCACTCGCCAGTGCAACTAC | CGGTCTTGAAGAAATGGTCT |
| *IFIT1* | AAGCTTGAGCCTCCTTGGGTTCGT | TCAAAGTCAGCAGCCAGTCTCAGG |
| *UPS18* | CAGACCCTGAACAATCCACCT | AGCTCATACTGCCCTCCAGA |
| *SOCS1* | AGAGCTTCGACTGCCTCTTC | CTCAGGTAGTCGCGGAGGAC |
| *SOCS2* | TAAAAGAGGCACCAGAAGGAAC | TCGATCAGATGAACCACACTG |
| *SOCS3* | GCCACCTACTGAACCCTCCT | ACGGTCTTCCGACAGAGATG |
| *GAPDH* | CATGAGAAGTATGACAACAGCCT | AGTCCTTCCACGATACCAAAGT |
