# Supplementary material for: Hepatitis E virus persists in the presence of a type III interferon response
Source: PLoS Pathog. 2017 May 30;13(5):e1006417. doi: 10.1371/journal.ppat.1006417 (PMC5466342; doi:10.1371/journal.ppat.1006417)
Supplement: S1 Fig — (DOCX) [file ppat.1006417.s002.docx]

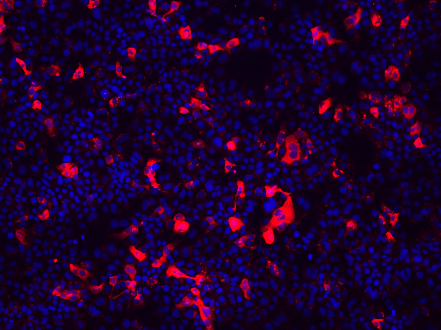

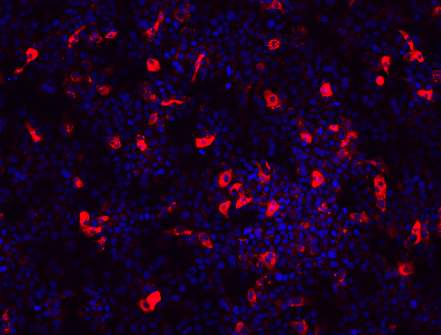

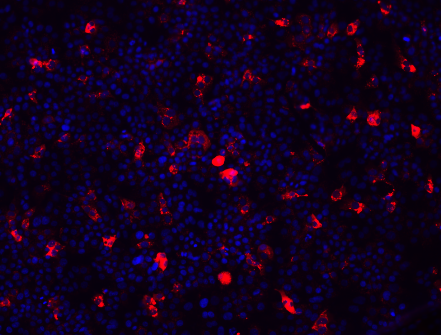

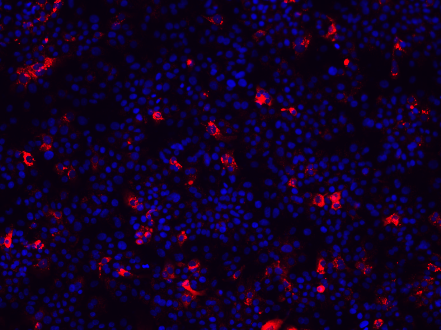

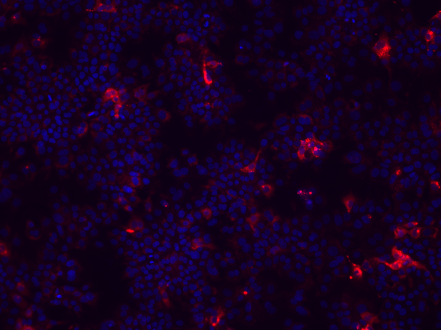

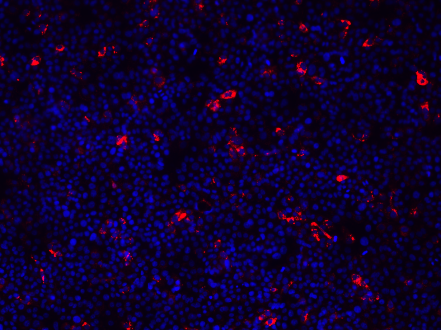


**d5**

**d10**

**d15**

**d20**

**d25**

**d30**

A

B

S1 Fig. Long-term HEV replication and IFN-λ production in HepG2 cells. HepG2 cells were inoculated with HEV (Kernow C1/P6) and split every 5 days. Virus-infected cells were determined by IFA (A) and supernatant IFN-λ was measured by ELISA (B) at indicated time points. Scale bar: 100 μm.
