# Supplementary material for: Hepatitis E virus persists in the presence of a type III interferon response
Source: PLoS Pathog. 2017 May 30;13(5):e1006417. doi: 10.1371/journal.ppat.1006417 (PMC5466342; doi:10.1371/journal.ppat.1006417)
Supplement: S2 Fig — (DOCX) [file ppat.1006417.s003.docx]

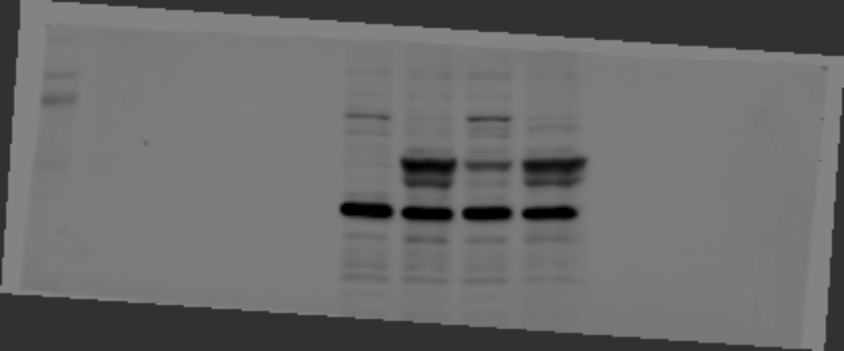

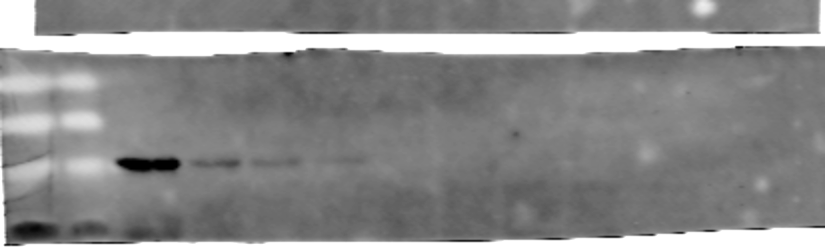

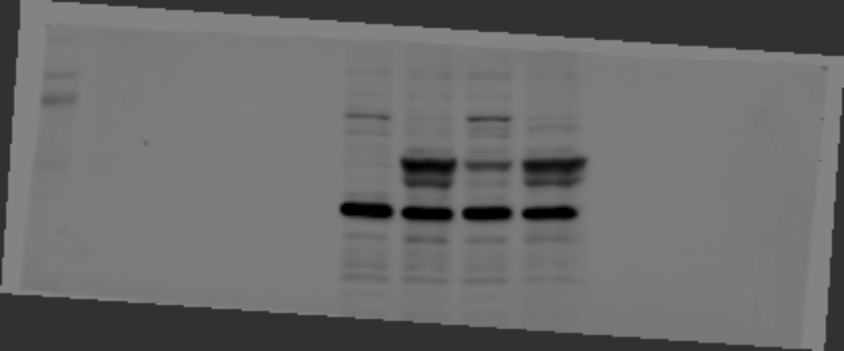


Recombinant

IFN-λ4 protein (ng)

100 50 25 12.5

IFN-λ4

ISG56

β-actin

Parental

Replicon

- + - +

Poly (I:C)

S2 Fig. Lack of IFN-λ4 protein production in HepG2 cells and HepG2 replicon cells. HepG2 or HepG2/replicon cells were transfected with poly IC (1.5 ug/mL). 12 hr post-transfection, cells were harvested and subjected to western blotting using antibodies against IFN-λ4, ISG56, and β-actin. Recombinant IFN-λ4 protein was included as a positive control.
