# Supplementary material for: Hepatitis E virus persists in the presence of a type III interferon response
Source: PLoS Pathog. 2017 May 30;13(5):e1006417. doi: 10.1371/journal.ppat.1006417 (PMC5466342; doi:10.1371/journal.ppat.1006417)
Supplement: S3 Fig — (DOCX) [file ppat.1006417.s004.docx]

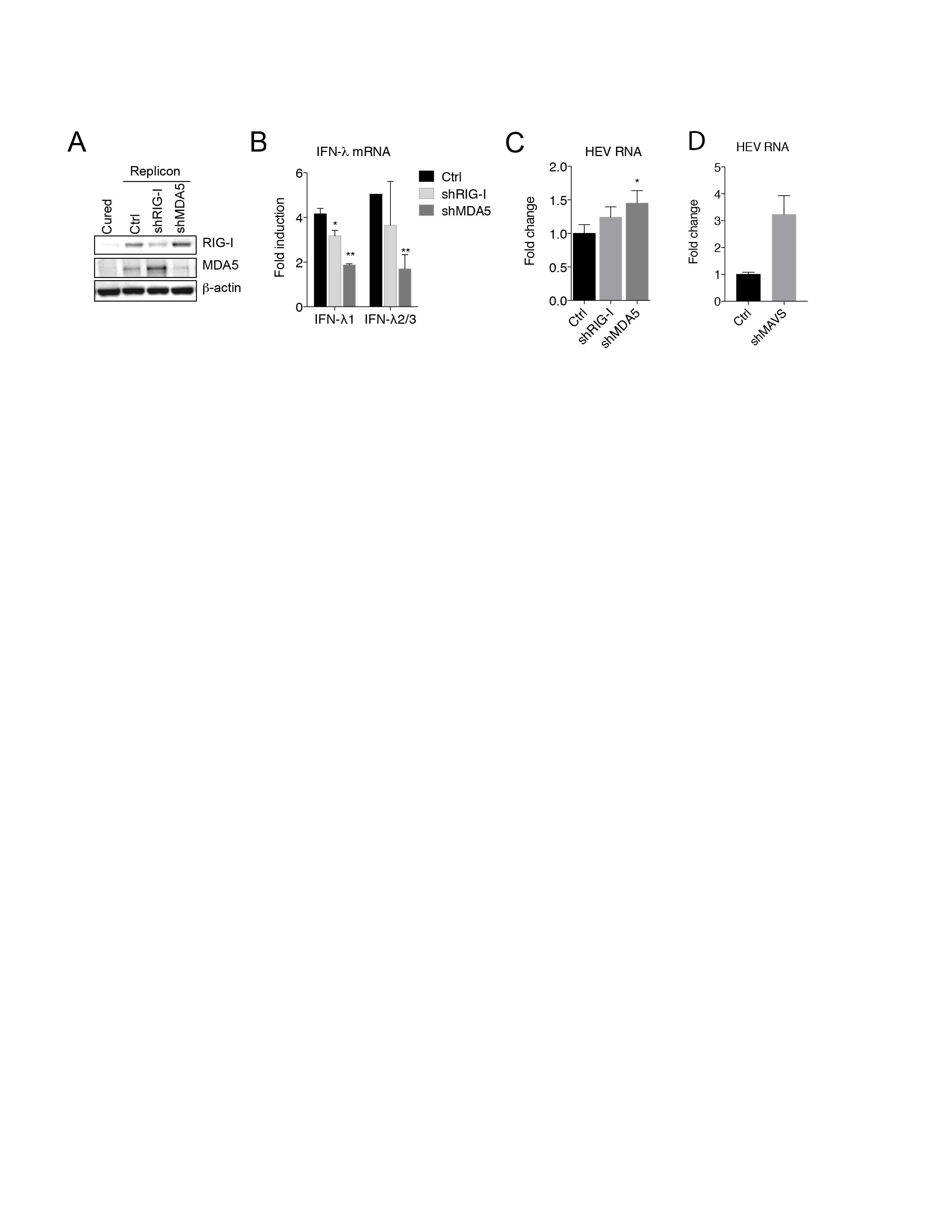


S3 Fig. Effect of RIG-I, MDA5, and MAVS knockdown on HEV replication in HepG2/replicon cells. **(A)** Immunoblots of RIG-I, MDA5 and β-actin in HEV replicon cells transduced with lentiviruses expressing GFP (Ctrl) or gene-specific shRNA. **(B)** IFN-λ mRNA expression in different HepG2 cells lines. (**C**) HEV RNA abundance in different HepG2 cell lines. (**D**) HEV RNA abundance in replicon cells and replicon cells transduced with lentivirus expressing MAVS-specific shRNA. The knockdown efficiency of MAVS was shown in the main Figure 5, panel F. The results show the mean ± SEM of 2 independent experiments performed in duplicate each. * P<0.05; ** P<0.01.
