# Supplementary material for: Hepatitis E virus persists in the presence of a type III interferon response
Source: PLoS Pathog. 2017 May 30;13(5):e1006417. doi: 10.1371/journal.ppat.1006417 (PMC5466342; doi:10.1371/journal.ppat.1006417)
Supplement: S4 Fig — (DOCX) [file ppat.1006417.s005.docx]

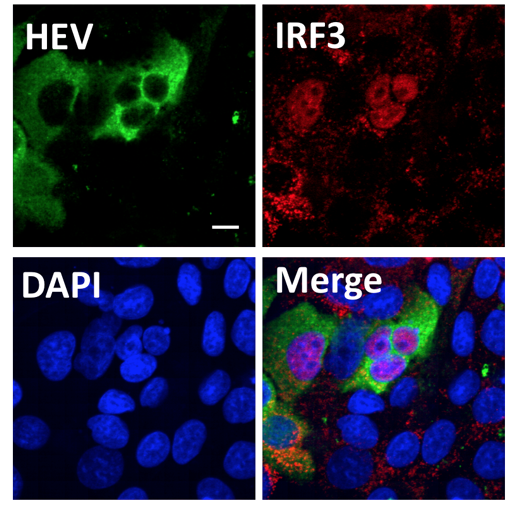


S4 Fig. IRF3 nuclear translocation in HEV-infected HepG2 cells. IRF3 was stained with a rabbit polyclonal antibody and HEV antigens were stained with chimpanzee serum ch1313. Scale bar: 10 μm.
