# Supplementary material for: Hepatitis E virus persists in the presence of a type III interferon response
Source: PLoS Pathog. 2017 May 30;13(5):e1006417. doi: 10.1371/journal.ppat.1006417 (PMC5466342; doi:10.1371/journal.ppat.1006417)
Supplement: S5 Fig — (DOCX) [file ppat.1006417.s006.docx]

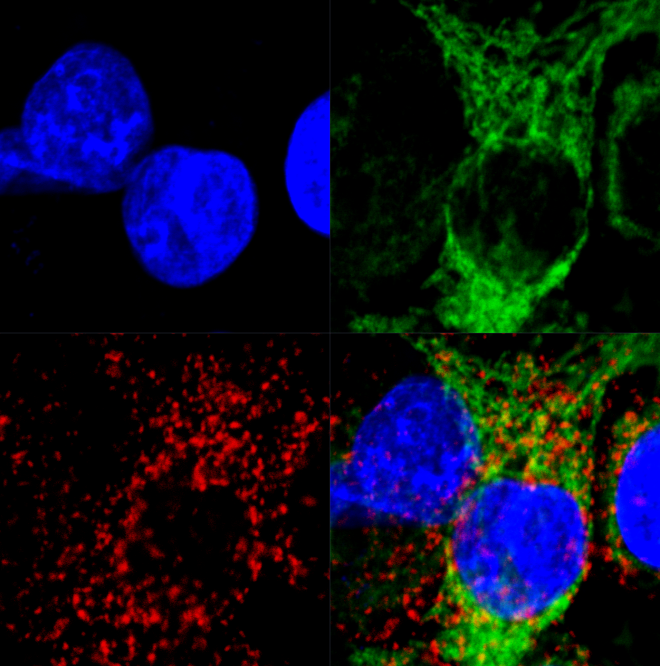

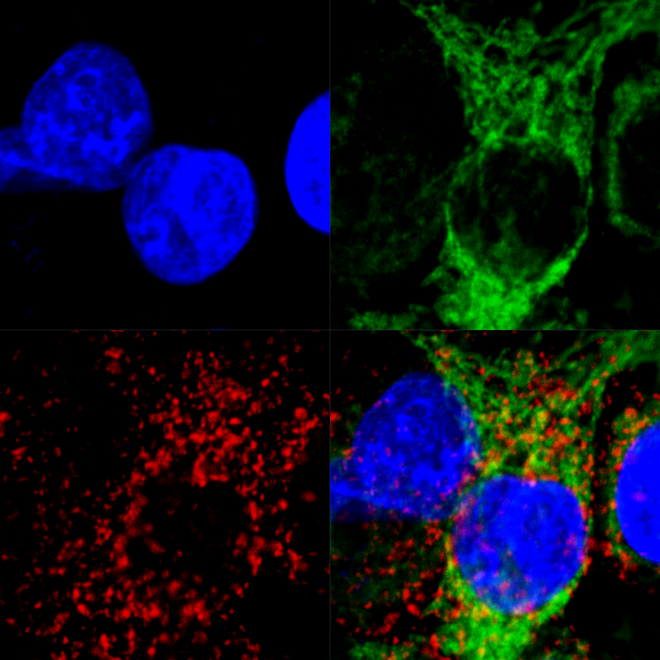

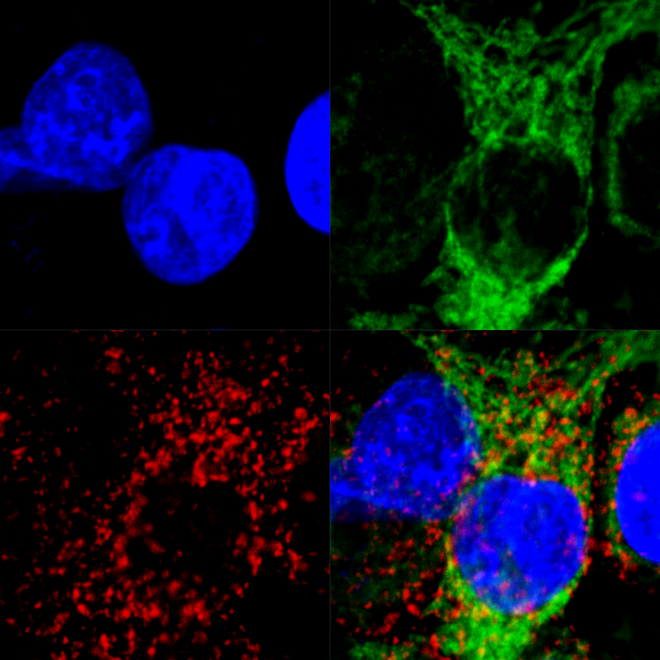

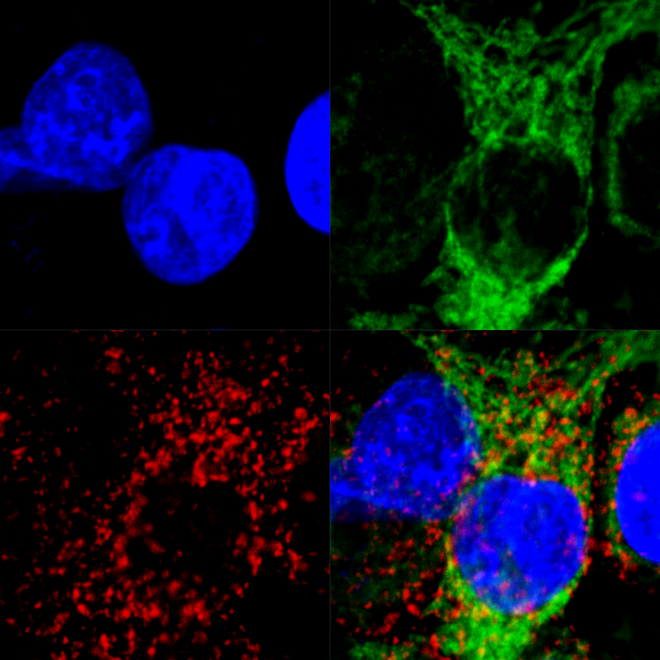

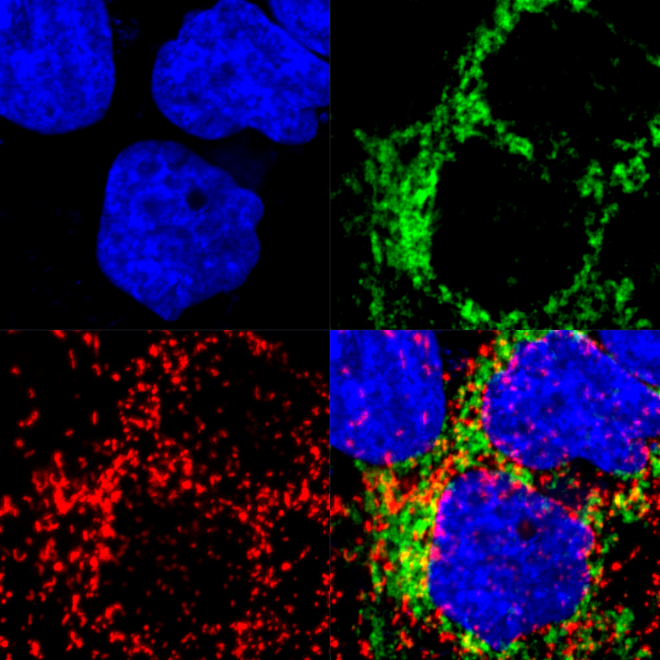

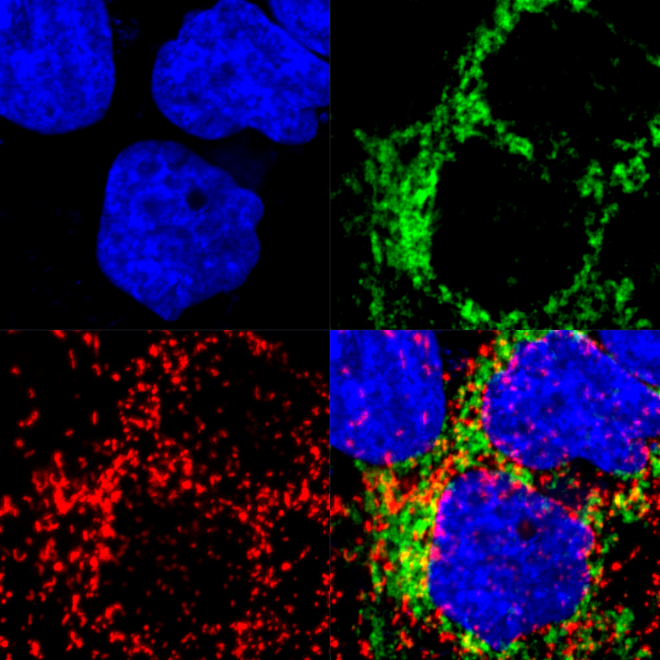

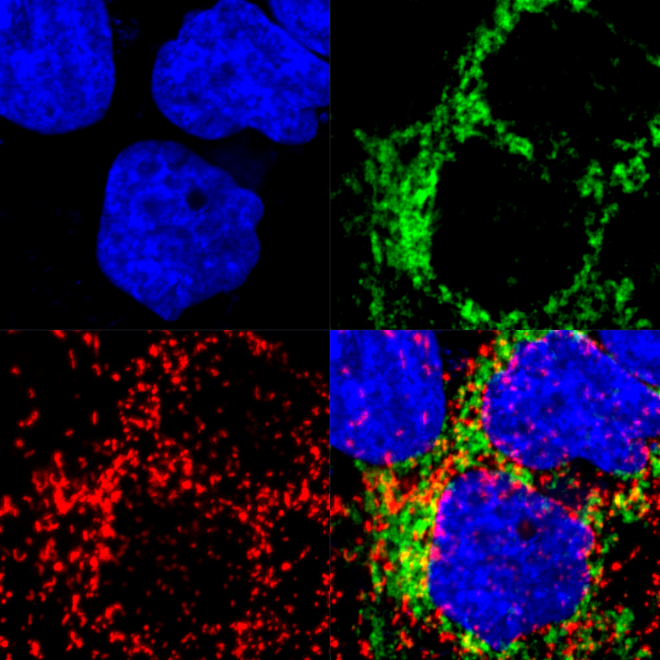

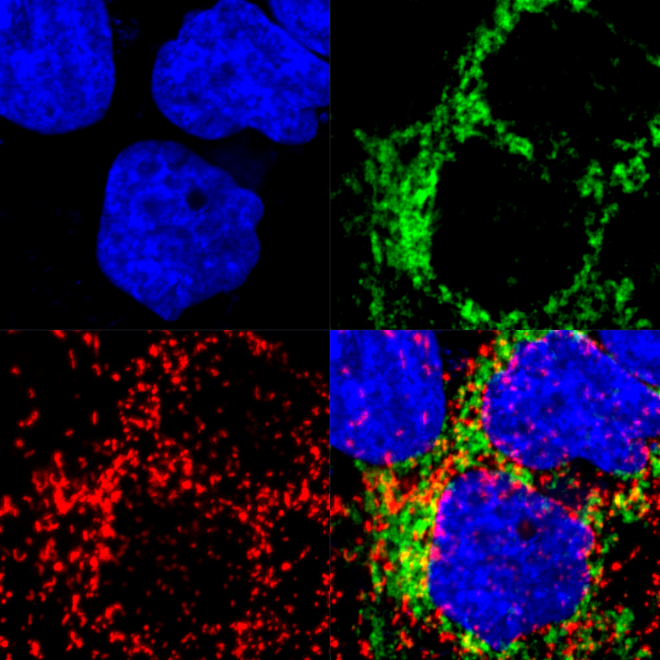


**MAVS**

**PMP70**

**DAPI**

**Merge**

HepG2

HepG2

replicon

S5 Fig. Confocal images showing the localization of MAVS and peroxisomes, stained with a rabbit antibody against MAVS (green) and a murine antibody against PMP70 (red), respectively. Scale bar: 10 μm.
