# Supplementary material for: Hepatitis E virus persists in the presence of a type III interferon response
Source: PLoS Pathog. 2017 May 30;13(5):e1006417. doi: 10.1371/journal.ppat.1006417 (PMC5466342; doi:10.1371/journal.ppat.1006417)
Supplement: S6 Fig — (DOCX) [file ppat.1006417.s007.docx]

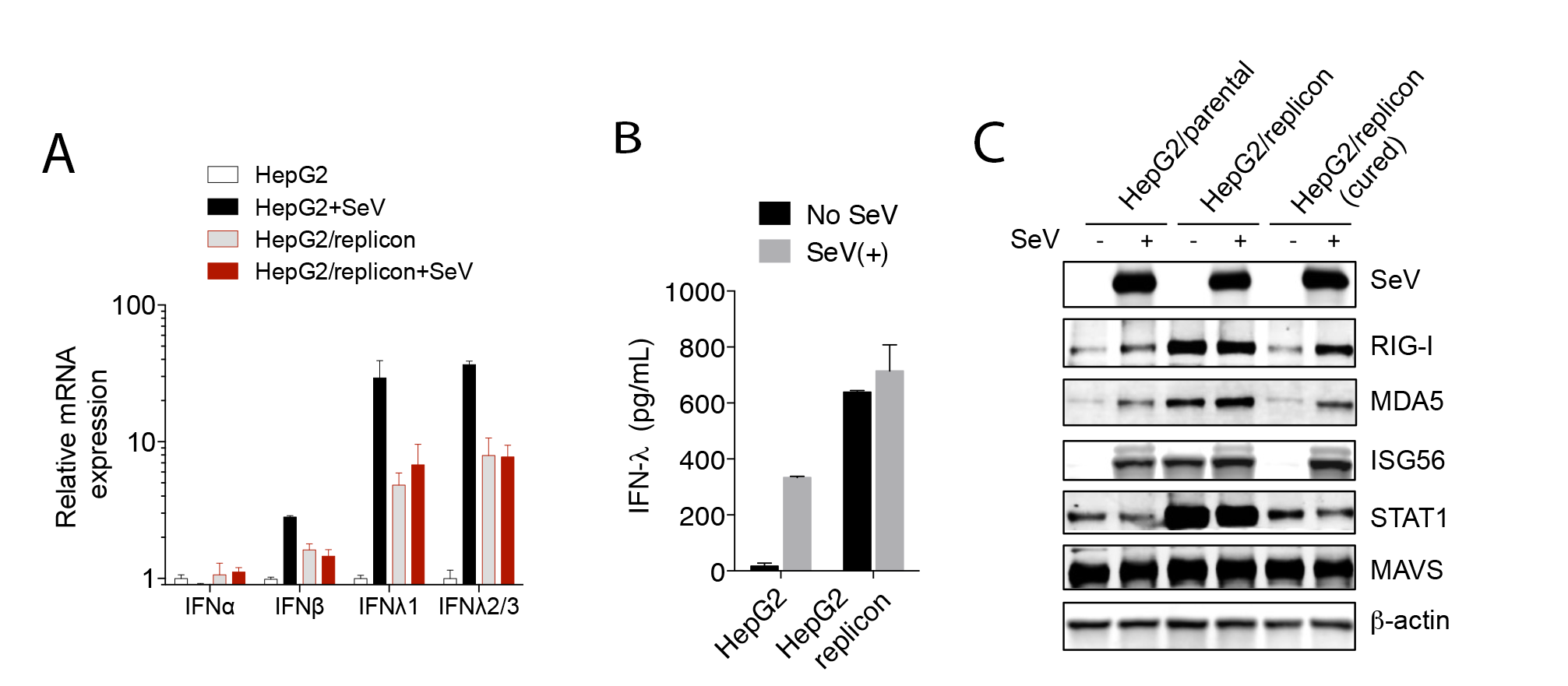

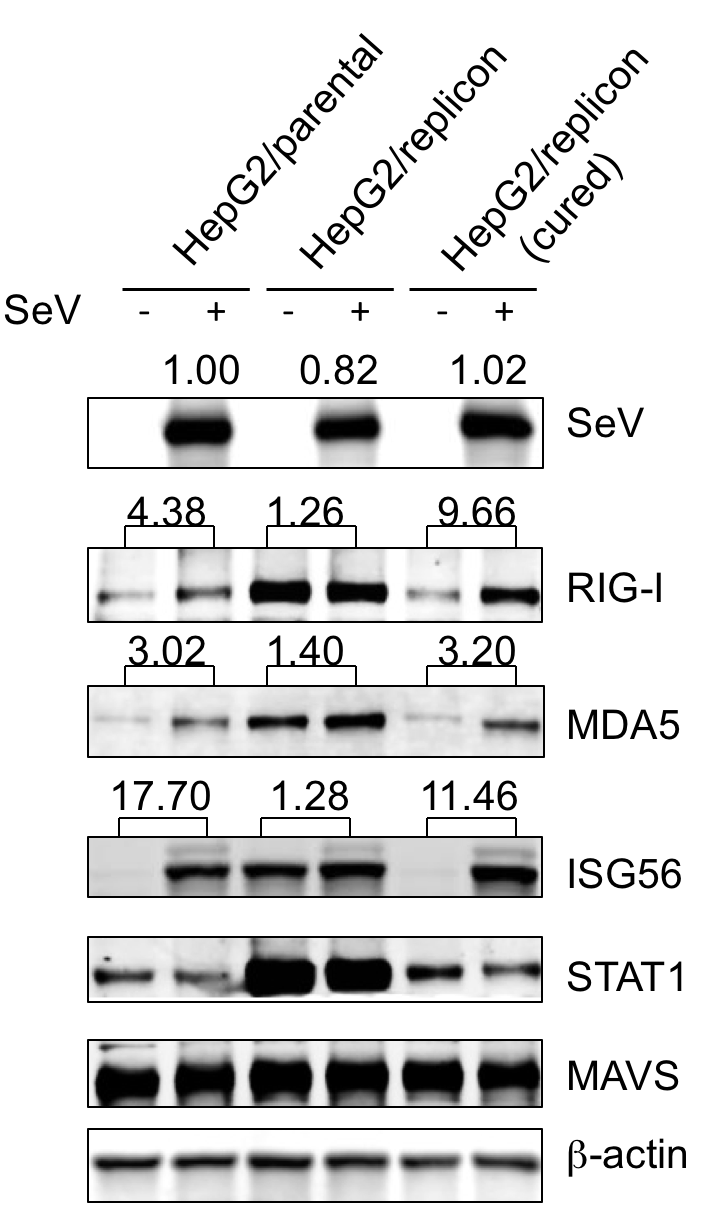


A

S6 Fig. Impaired RIG-I signaling in HepG2 cells harboring an HEV replicon. (**A**) HepG2 cells with or without the HEV replicon were infected with SeV for 16 hr. Intracellular IFN mRNA levels were measured by qRT-PCR. Data are expressed as fold induction relative to mock infected cells containing no replicon. Each data point represents the mean ± SEM of 2 independent experiments in duplicate each. *, p<0.05; **, p<0.01. (**B**) Supernatants from (A) were assayed for IFN-λ proteins. The results show the mean ± SEM of 2 independent experiments. **(C)** Immunoblots of SeV, RIG-I, MDA5, ISG56, STAT1, MAVS and β-actin in different HepG2 cells (parental, replicon-containing, and replicon-cured) before or after SeV infection (16 h). Bands were quantified by the LI-COR imaging software. Numbers above the blots for RIG-I, MDA5 and ISG56 indicate fold changes relative to uninfected cells. Numbers above the blot for SeV indicate fold changes relative to that in the parental cells.
