# Supplementary material for: Hepatitis E virus persists in the presence of a type III interferon response
Source: PLoS Pathog. 2017 May 30;13(5):e1006417. doi: 10.1371/journal.ppat.1006417 (PMC5466342; doi:10.1371/journal.ppat.1006417)
Supplement: S7 Fig — (DOCX) [file ppat.1006417.s008.docx]

S7 Fig. Effect of HEV on IFN-induced ISG expression. HepG2 cells with or without the HEV replicon were treated with IFN-α or IFN-λ for 6 h. Endogenous ISG15 and IFIT1 mRNA levels were measured by qRT-PCR. Shown are representative results (mean ± SEM) from one of two independent experiments.
