# Supplementary material for: Hepatitis E virus persists in the presence of a type III interferon response
Source: PLoS Pathog. 2017 May 30;13(5):e1006417. doi: 10.1371/journal.ppat.1006417 (PMC5466342; doi:10.1371/journal.ppat.1006417)
Supplement: S8 Fig — (DOCX) [file ppat.1006417.s009.docx]

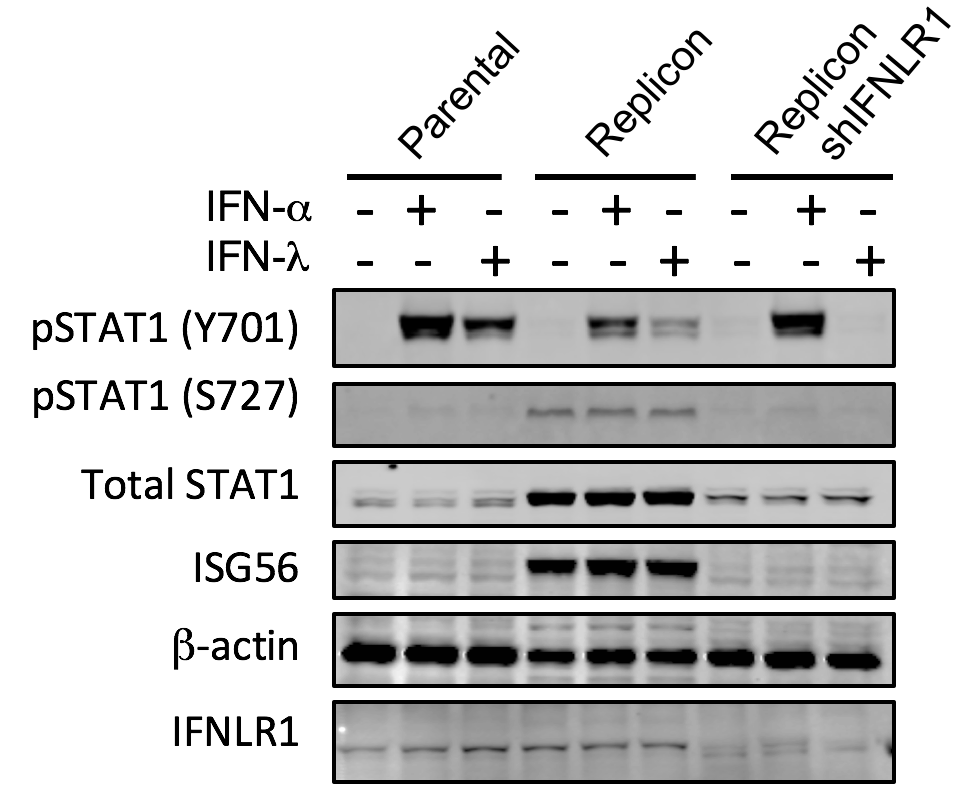


S8 Fig. IFNLR1 depletion restores IFN-induced STAT1 phosphorylation in the HepG2 replicon cells. Cells were treated with recombinant IFN-α (100 ng/mL) or IFN-λ (220 ng/ml) for 1 h and subjected to Western blotting with indicated antibodies.
