# Supplementary material for: Hepatitis E virus persists in the presence of a type III interferon response
Source: PLoS Pathog. 2017 May 30;13(5):e1006417. doi: 10.1371/journal.ppat.1006417 (PMC5466342; doi:10.1371/journal.ppat.1006417)
Supplement: S9 Fig — (DOCX) [file ppat.1006417.s010.docx]

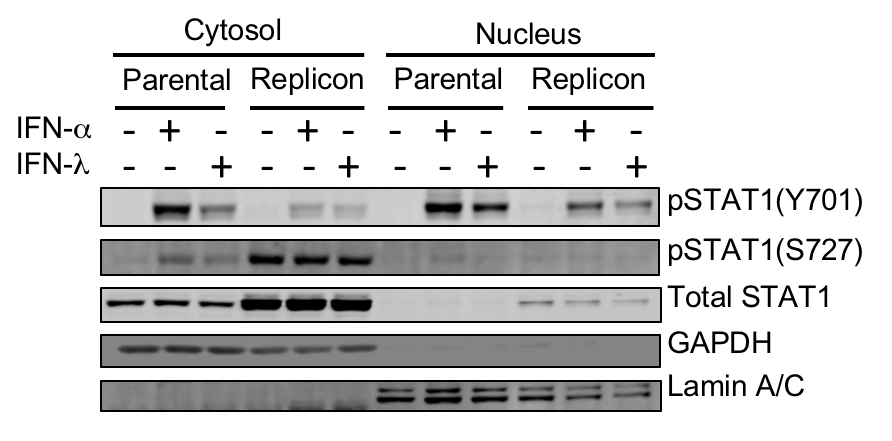


S9 Fig. Distribution of STAT1 and pSTAT1 between cytosol and nucleus in different HepG2 cells. Cells were treated with IFN-α (100 ng/ml) or IFN-λ (220 ng/ml) or mock treated for 1 h. Cytosolic and nuclear fractions were separated using the NE-PER Nuclear and Cytoplasmic Extraction Reagents (Cat#78833, Thermo Scientific, Inc.) according to manufacturer’s protocol. The cytoplasmic and nuclear fractions were then subjected to Western blot analysis using indicated antibodies. GAPDH was used as a marker for the cytosol, and Lamin A/C was used as a marker for the nucleus.
