# Supplementary material for: Hepatitis E virus persists in the presence of a type III interferon response
Source: PLoS Pathog. 2017 May 30;13(5):e1006417. doi: 10.1371/journal.ppat.1006417 (PMC5466342; doi:10.1371/journal.ppat.1006417)
Supplement: S10 Fig — (DOCX) [file ppat.1006417.s011.docx]

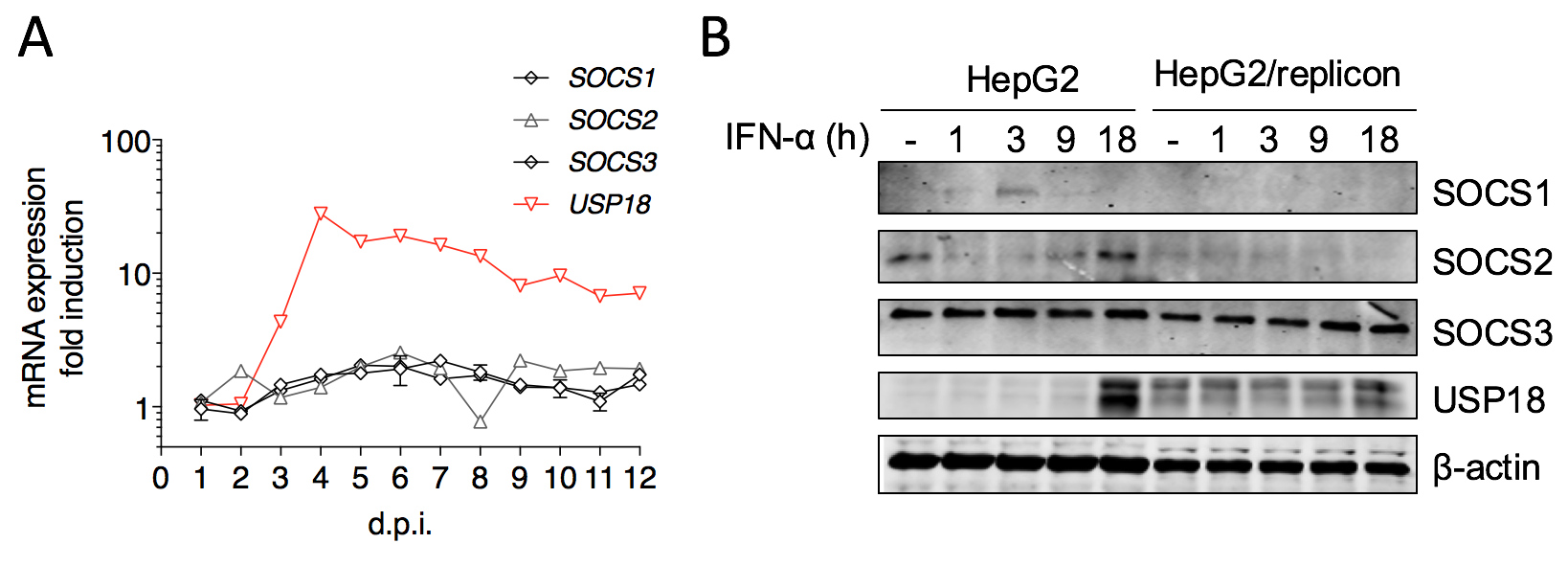


S10 Fig. (A) SOCS1-3 and USP18 mRNA expression in HepG2 cells following HEV infection. (B) SOCS1-3 and USP18 protein expression in HepG2 and HepG2/replicon cells before and after treatment with IFN-α (100 ng/ml) for indicated time.
