# Supplementary material for: Hepatitis E virus persists in the presence of a type III interferon response
Source: PLoS Pathog. 2017 May 30;13(5):e1006417. doi: 10.1371/journal.ppat.1006417 (PMC5466342; doi:10.1371/journal.ppat.1006417)
Supplement: S11 Fig — (DOCX) [file ppat.1006417.s012.docx]

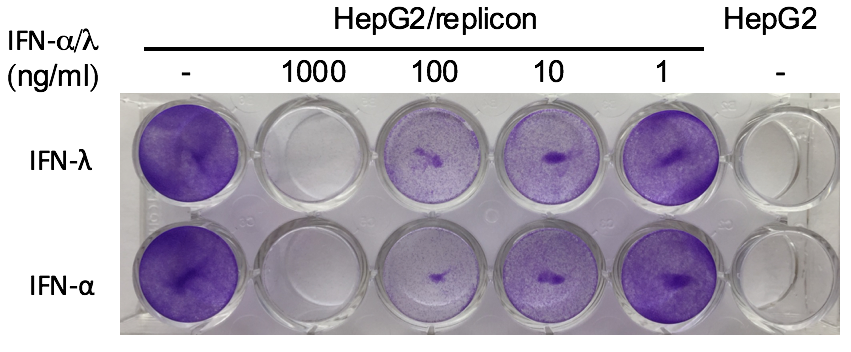


S11 Fig. Elimination of HEV replicon RNA following prolonged treatment with high doses of IFNs. HepG2 cells containing the HEV replicon were treated with IFN-α or IFN-λ at indicated concentrations every 3 days for 18 days in the absence of G418. Equal numbers of treated cells and HepG2 cells without the replicon (5x10^4^ cells/well) were seeded into a new 24 well plate and grown in the presence of G418 (400 μg/ml) for another 6 days before they were fixed and stained with crystal violet.
